# Lung function in children with cystic fibrosis in the USA and UK: a comparative longitudinal analysis of national registry data

**DOI:** 10.1136/thoraxjnl-2021-216849

**Published:** 2021-05-11

**Authors:** Daniela K Schlüter, Josh S Ostrenga, Siobhán B Carr, Aliza K Fink, Albert Faro, Rhonda D Szczesniak, Ruth H Keogh, Susan C Charman, Bruce C Marshall, Christopher H Goss, David Taylor-Robinson

**Affiliations:** 1 Department of Public Health, Policy and Systems, University of Liverpool, Liverpool, UK; 2 Cystic Fibrosis Foundation, Bethesda, Maryland, USA; 3 Royal Brompton Hospital, London, UK; 4 National Heart and Lung Institute, Imperial College London, London, UK; 5 Department of Pediatrics, Cincinnati Children's Hospital Medical Center, Cincinnati, Ohio, USA; 6 Department of Medical Statistics, London School of Hygiene and Tropical Medicine, London, UK; 7 UK Cystic Fibrosis Trust, London, UK; 8 Departments of Medicine and Pediatrics, University of Washington, Seattle, Washington, USA

**Keywords:** cystic fibrosis, paediatric lung disaese, clinical epidemiology

## Abstract

**Rationale:**

A previous analysis found significantly higher lung function in the US paediatric cystic fibrosis (CF) population compared with the UK with this difference apparently decreasing in adolescence and adulthood. However, the cross-sectional nature of the study makes it hard to interpret these results.

**Objectives:**

To compare longitudinal trajectories of lung function in children with CF between the USA and UK and to explore reasons for any differences.

**Methods:**

We used mixed effects regression analysis to model lung function trajectories in the study populations. Using descriptive statistics, we compared early growth and nutrition (height, weight, body mass index), infections (*Pseudomonas aeruginosa*, *Staphylococcus aureus*) and treatments (rhDnase, hypertonic saline, inhaled antibiotics).

**Results:**

We included 9463 children from the USA and 3055 children from the UK with homozygous F508del genotype. Lung function was higher in the USA than in the UK when first measured at age six and remained higher throughout childhood. We did not find important differences in early growth and nutrition, or *P.aeruginosa* infection. Prescription of rhDNase and hypertonic saline was more common in the USA. Inhaled antibiotics were prescribed at similar levels in both countries, but Tobramycin was prescribed more in the USA and colistin in the UK. *S. aureus* infection was more common in the USA than the UK.

**Conclusions:**

Children with CF and homozygous F508del genotype in the USA had better lung function than UK children. These differences do not appear to be explained by early growth or nutrition, but differences in the use of early treatments need further investigation.

**Key messages**

**What is the key question?**

- Was there a difference in the longitudinal trajectories of lung function in children with cystic fibrosis between the USA and UK during 2003–2014?

**What is the bottom line?**

- Children with cystic fibrosis and homozygous F508del genotype in the UK had persistently worse lung function than those in the USA.

**Why read on?**

- This finding does not appear to be explained by differences in casemix or early growth or nutrition, but there are differences in the use of early treatments between the two countries which will need further investigation.

## Introduction

Cystic fibrosis (CF) is a serious, multiorgan inherited disease characterised by pulmonary infections and progressively declining lung function. Most people with CF die prematurely from their disease through respiratory failure.^1 2^ In the 1960s, median survival in the UK was estimated to be below 10 years of age.^3^ Over the past decades, outcomes have improved due to multidisciplinary care, nutritional support and new treatments, such that half of the babies born with CF in the UK and the USA today can be expected to live at least in to their late forties.^2 4–6^

Previous international comparisons of outcomes in people with CF have highlighted the impact of different healthcare practices and approaches to treatment, and have contributed to improvements in care for people with CF. For example, comparisons of nutritional, pulmonary and survival outcomes between the USA and Canadian CF populations provided evidence for high-fat, high-calorie diets for people with CF.^7 8^

A previous cross-sectional study comparing the 2010 US and UK CF populations suggested better lung function in children in the USA compared with those in the UK. After adjustment for a set of potential demographic and clinical confounders, the authors estimated a difference in percent of predicted forced expiratory volume in 1 s (%FEV_1_) of 7.62 percentage points (95% CI 6.24 to 9.00) between children in the USA and UK under the age of 12. This difference seemed to be smaller in adolescence and disappeared by age 30.^9^ However, due to the cross-sectional nature of the study, the age trends demonstrated could not be interpreted as average trends for individuals over time. The narrowing of the US-UK gap with increasing age was therefore difficult to interpret, conflating potentially different rates in lung function decline between populations with cohort effects and survivor bias later in adulthood. The aim of this study was, therefore, to compare lung function trajectories, that is, lung function at age 6 years and rates of lung function decline, between the US and UK populations. Secondary aims were to explore potential reasons for any differences demonstrated. To ensure comparability of populations, we restricted our analysis to the paediatric populations to reduce the impact of survivor bias, and to people who were homozygous F508del—the most common CF genotype—to reduce differences in case-mix.

## Methods

### Study design

We carried out retrospective longitudinal analyses of lung function in comparable cohorts of children with CF aged ≥6 to<18 years and with homozygous F508del genotype captured in the US Cystic Fibrosis Foundation Patient Registry (CFFPR) and UK Cystic Fibrosis Registry.

### Data sources

We used the US CFFPR and the UK CF Registry. The current CFFPR includes data from 1986 onwards on over 50 000 people with CF and is estimated to capture about 84% of the current US CF population. Since 2003, CF care centres have been encouraged to enter information from all clinical encounters at CF Foundation Care Centre Network facilities.^10^ Current CFF guidelines are for people with CF to be seen quarterly for routine care.^11^

In the UK, it is recommended that care teams submit annual encounter data to the Registry from a clinic visit approximately 12 months after the previous entry and when the patient is clinically stable. Records date back to the 1990s and are estimated to capture approximately 99% of the current CF population.^12^ See online supplemental material section 1 for more details.

10.1136/thoraxjnl-2021-216849.supp1 Supplementary data

### Setting and participants

We included children aged ≥6 to <18 with homozygous F508del genotype with data recorded in the CFFPR or UK CF Registry between January 2003 and December 2014 and with at least one lung function measurement in that time period. Restricting the analysis to children with CF who were homozygous for F508del reduced the differences in casemix between the two countries. The dates were chosen to ensure comparability between populations as it preceded licensing of Orkambi (lumacaftor/ivacaftor) in the USA. For individuals who had a transplant, observations were censored at transplant.

We excluded individuals who did not have complete data on sex, year of birth and age at diagnosis.

### Outcomes and covariates

Our main outcome of interest was lung function measured by percent of predicted %FEV_1_ based on GLI reference equations^13^ and the rate of lung function decline.

Secondary outcomes were time-varying indicators of growth and nutrition (height, weight, body mass index (BMI); raw values and z-scores using the Centers for Disease Control and Prevention (CDC) reference populations in both countries and additionally UK-WHO reference population in the UK), prescribed chronic medications (rhDNase, hypertonic saline), common inhaled antibiotics (Tobramycin, Colistin, Aztreonam) and common infections (*Pseudomonas aeruginosa*, *Staphylococcus aureus*) between birth and age 18 for the study population. More information on secondary outcomes and when they began collection can be found in online supplemental material section 1 table 1.

To adjust for remaining potential differences in case-mix, we included the following baseline covariates in the models used: sex (binary: male/female; reference level: female), year of birth (continuous, centred at 1997) and age at diagnosis (continuous).

### Statistical analysis

Using all the collected data on our study population from age 6 up to (excluding) age 18 (encounter data in the USA and annual review data in the UK), we developed models for the longitudinal trajectories of lung function to compare estimated population-level mean lung function and population-level mean lung function decline by age between the US and UK paediatric populations.

We fitted a series of models of different complexity to the US and UK study populations. We fitted models using linear,^14 15^ quadratic and cubic polynomials of age, and models including flexible functions of age^16 17^ using natural cubic splines with one knot at age 12, two knots at ages 8 and 14, 5 knots with one knot every 2 years (8, 10, 12, 14 and 16 years) and 11 knots – one every year. All models included sex, year of birth and age at diagnosis as covariates; year of birth and age at diagnosis entered the models linearly. We did not consider any interactions between functions of age and the covariates (for exploratory plots, see online supplemental material section 2).

To appropriately capture the correlation between repeated measurements within an individual over time, we initially included random intercepts in the models and then added: (1) random slope (only in the model with a linear function of age), (2) random slope and exponential correlation function (only in the model with a linear function of age), (3) exponential correlation function and (4) linear correlation function. Within each country all the model fits were compared using the log-likelihood, Akaike information criterion (AIC) and Bayesian information criterion (BIC); the best fitting models are those with the highest log-likelihood, lowest AIC and lowest BIC. For more details, see online supplemental material section 3.

To explore reasons for any differences in lung function trajectories we compared indicators of growth and nutrition, treatments, and infections cross-sectionally from birth to age 18 between the two study populations. This included records collected prior to 2003 on individuals in the study population. Observations remained censored at lung transplant. We summarised height, weight and BMI by their median, 25th and 75th percentile at each year of age for all individuals in the study population for whom data were available at that age. For treatments and infections we presented the proportion of individuals with at least one record of receiving the treatments/having the infections before age 6, before age 12 and before age 18 years; as well as the median, 25th and 75th percentile, minimum and maximum of the age at first recorded treatment/infection.

We stratified these comparisons by year of birth (before and after 1997) as post 1997 data will be more complete with regard to treatments and infections and may give a better representation of healthcare practices.

We used R packages nlme^18^ and splines^19^ for data analysis and ggplot2^20^ and the *dns*() function from the JMbayes^21^ package for visualisations; online supplemental material used knitr.^22 23^

### Robustness test

To assess the generalisability of our results to more recent paediatric CF populations, we repeated our analysis using the best fitting models applied to the recent cohort born after 1997.

## Results

### Study populations

There were 9463 and 3055 individuals in the US and UK study population, respectively (see online supplemental material section 4 for the derivation of the study populations). In the USA, individuals had a median of 24 lung function measurements, with a median of 4 (IQR: 3–6) measures per individual per year (table 1). In the UK, each individual had a recorded review measurement approximately once a year, with a median of 4 lung function measurements during the study period. The average lung function for those observed at age 6 was 93 (SD: 17.7) in the USA and 88.2 (SD: 16.9) %FEV_1_ in the UK. The populations were generally comparable with regard to their demographic characteristics (table 1, online supplemental material section 5).

**Table 1:** Details of the datasets and study populations in the USA and UK, respectively

	US data	UK data
Sample size	9463	3055
No of FEV1 measures per person (median (IQR), (full range))	24 (11–40), (1–199)	4 (2–7), (1–17)
No of individuals with one FEV_1_ measure (%)	251 (2.7)	398 (13.0)
No of FEV_1_ measures per person per year (median (IQR))	4 (3–6)	1 (1–1)
Time in years between first and last FEV_1_ measurement included in the study per person (median (IQR))*	5.5 (2.7–8.4)	4.9 (2.2–7.5)
Informative drop-out during study period (%)		
Lung transplants	142 (1.5)	27 (0.9)
Deaths	263 (2.8)	70 (2.3)
Sex (%)		
Female	4625 (48.9)	1457 (47.7)
Male	4838 (51.1)	1598 (52.3)
Year of birth (median) (IQR)	1996 (1991–2002)	1996 (1991–2002)
Race (%)		
Non-Caucasian	384 (4.1)	71 (2.3)
Caucasian	9083 (95.9)	2984 (97.7)
Age at diagnosis in years (median (IQR))	0.3 (0.0–1.2)	0.2 (0.0–1.0)
Diagnosis by newborn screening (%)	1079 (11.4)	501 (16.3)
%FEV_1_ at age 6 (mean (SD))	92.96 (17.7)	88.17 (16.9)

*Excluding individuals with only one FEV1 measurement.

FEV_1_, forced expiratory volume in 1 s.

### Estimated lung function and lung function decline

In both countries, the estimated population-level mean lung function between ages 6 and 18 and the covariate effects were very similar across all the different models (online supplemental material section 6). The best fitting model for lung function in both countries was the model with a linear term for age with random intercept, random slope and exponential correlation function. In the USA, the model including age using a spline with five knots, random intercept and exponential correlation function gave only a marginally worse fit, therefore, we report results for both models.

**Figure 1:**
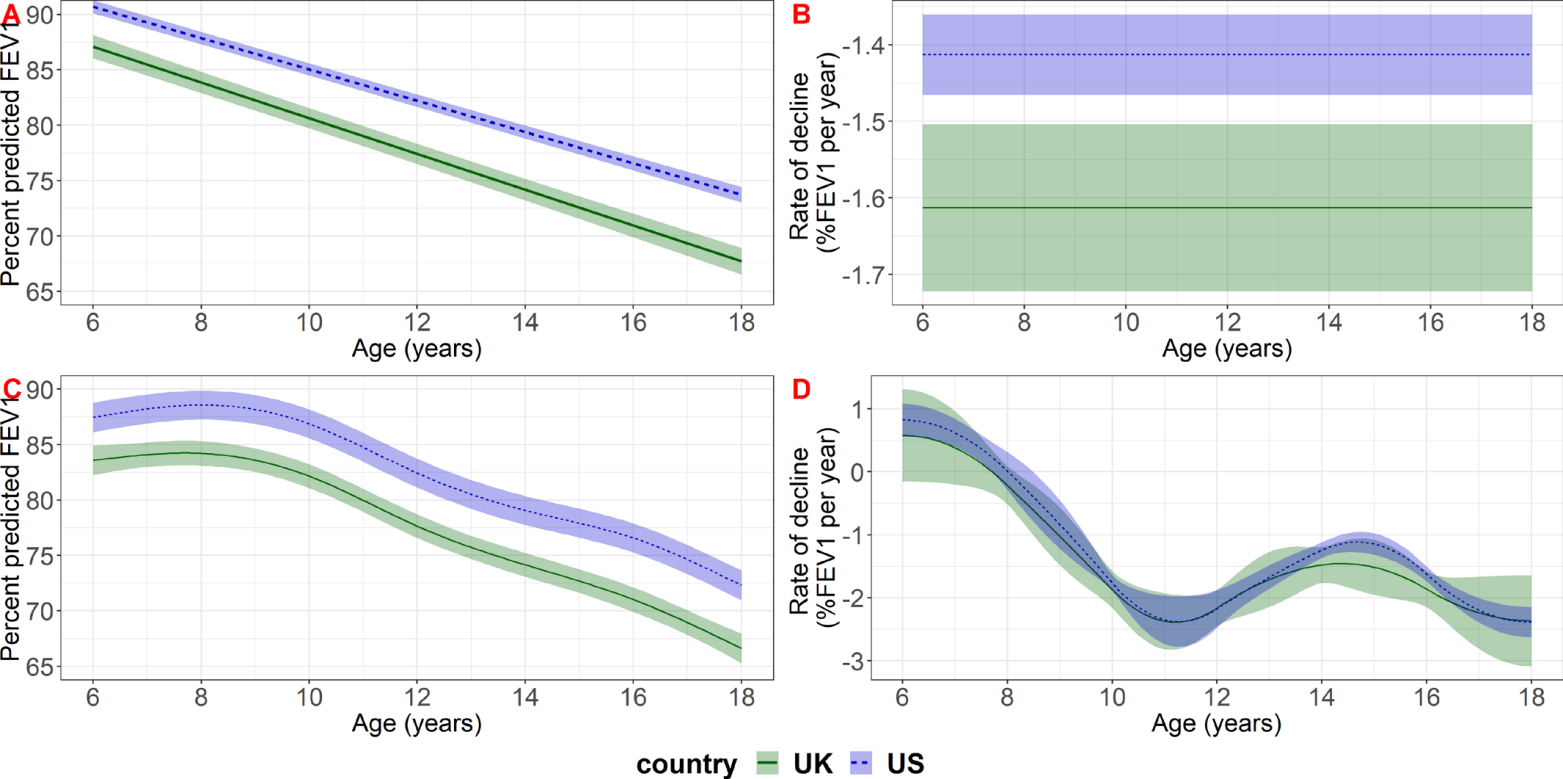
Estimated population-level mean lung function (panels A, C) and lung function decline (panels B, D) in the UK and US study populations for reference covariate values (female, born in 1997, diagnosed at birth). The top row (A, B) shows results based on the model that included a linear term for age with random intercept and slope and exponential correlation function; the bottom row (C, D) shows results based on the model that included age using a spline with five knots with random intercept and exponential correlation function. FEV, forced expiratory volume in 1 s.

Population mean lung function was higher in the US study population throughout childhood (figure 1). Based on our model that included a linear function of age, lung function declined at a faster rate in the UK than in the US population (−1.61 per year (95% CI: −1.72 to −1.50) in the UK compared with −1.41 (95% CI: −1.47 to –1.36) in the USA); children in the UK lost on average an additional 0.20 percentage points in %FEV_1_ per year (95% CI 0.08 to 0.32) compared with the USA. Based on the model that included age using a spline with five knots there was an indication of a faster rate of decline in the UK at almost all ages but the evidence was not clear (figure 1, online supplemental material section 8).

Based on the model with a linear term for age and covariate reference levels (female, born in 1997, diagnosed at birth) the estimated difference between the USA and UK populations at ages 6, 12 and 17 was 3.60 (95% CI 2.40 to 4.80), 4.80 (95% CI 3.38 to 6.22) and 5.80 (95% CI 3.98, 7.62) % FEV_1_, respectively. Based on the model including age using a spline model with five knots, the difference between the two countries was 3.89 (95% CI 2.00, 5.77), 4.78 (95% CI 3.10 to 6.47) and 5.69 (95% CI 3.97 to 7.41)% FEV_1_ at ages 6, 12 and 17, respectively. The US-UK gap was estimated to increase over calendar time by 0.13% predicted per year of birth (95% CI 0.003 to 0.25) based on the model with a linear term for age, and 0.12% predicted per year (95% CI −0.01 to 0.25) based on the model including age using a spline with five knots (table 2). In the UK, males had on average 2.50 percentage points (95% CI 1.32 to 3.67) higher %FEV_1_ than females, whereas in the USA, the difference was 1.42 percentage points (95% CI 0.70 to 2.14) (based on the model with a linear term for age; results were similar for the model that included age using a spline with five knots). The difference between the US and UK populations may therefore be less for males than females (see online supplemental material section 7 for estimated differences between the US and UK for selected sets of covariate values).

**Table 2:** Estimated covariate effects (95% CIs) based on the model that included a linear term for age with random intercept, random slope and exponential correlation function and the model that included age using a spline with five knots, random intercept and exponential correlation function

	US		UK	
	Linear function	Spline	Linear function	Spline
Sex=male	1.42 (0.70, 2.14)	1.80 (1.04, 2.55)	2.5 (1.33, 3.7)	2.71 (1.49, 3.93)
Age at diagnosis	0.59 (0.43, 0.74)	0.57 (0.42, 0.72)	0.62 (0.30, 0.95)	0.6 (0.28, 0.92)
Year of birth	0.37 (0.30, 0.43)	0.42 (0.35, 0.48)	0.24 (0.13, 0.35)	0.3 (0.19, 0.41)

### Indicators of growth and nutrition, treatments and infections

**Figure 2:**
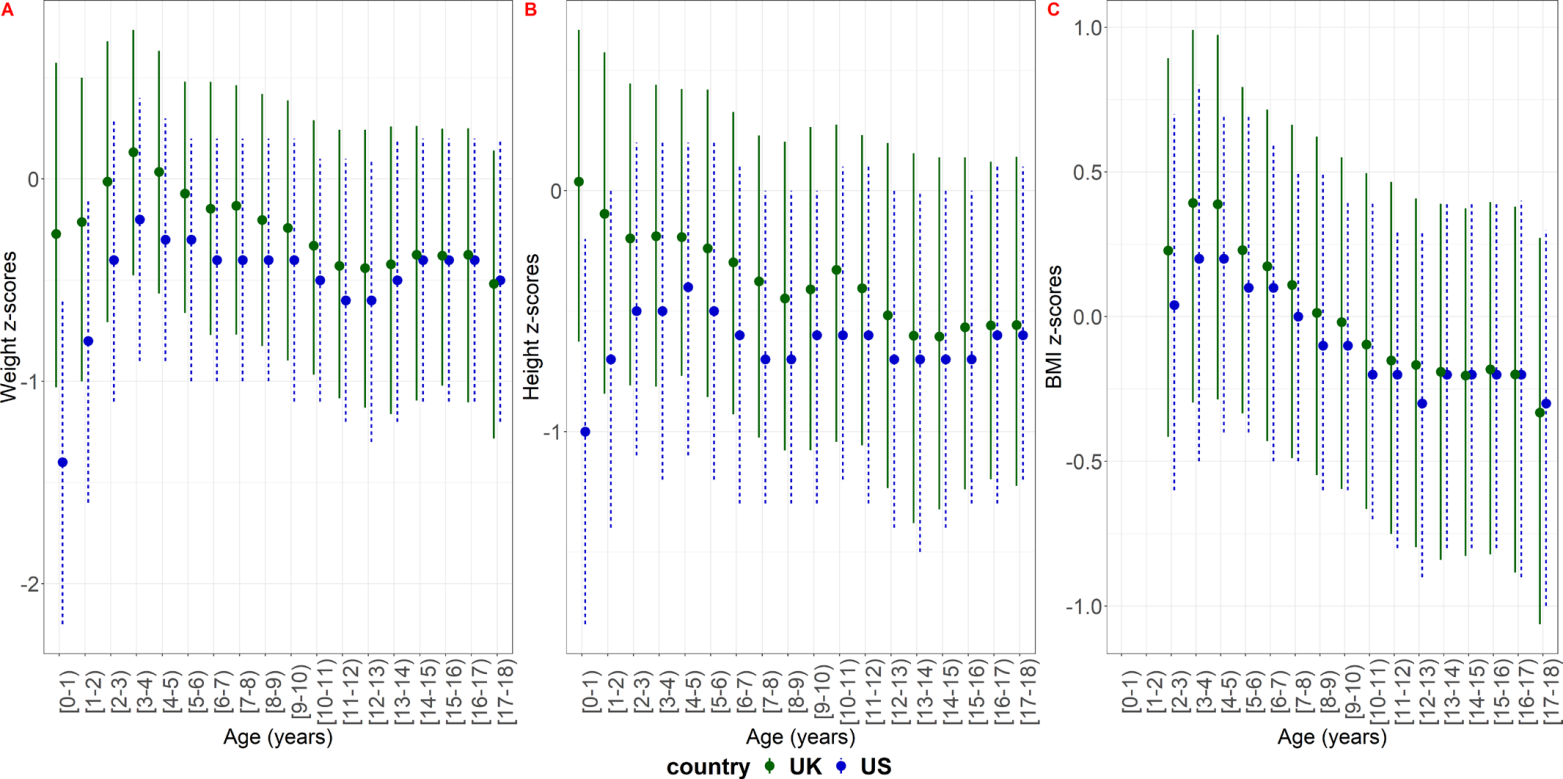
Cross-sectional summary measures of weight, height and BMI z-scores of the study population based on the CDC reference population in the US and the UK. The dots show the median and the lines the IQR. BMI, body mass index.

Children in the US study population were on average lighter and shorter than children in the UK population during childhood and into adolescence (figure 2, online supplemental material section 9).

Rates of infection with *P. aeruginosa* were similar in both countries but methicillin sensitive and methicillin resistant *S. aureus* infections were more common in the US (online supplemental material section 11).

A larger proportion of the study population was treated with rhDNase and hypertonic saline in the USA compared with the UK. In the post 1997 birth cohort, 73% were treated with rhDNase before age 6 in the USA and 96% before age 18 compared with 20% and 86%, respectively, in the UK (online supplemental material section 10). Hypertonic saline was prescribed for 76% of the study population before age 18 in the post 1997 birth cohort in the USA vs 50% in the UK (online supplemental material section 10). Inhaled antibiotics were prescribed at slightly higher levels before age 6 in the USA but prescribed at similar levels at later ages (online supplemental material section 10). However, there was a big difference in the inhaled antibiotic of choice between the two countries. Tobramycin was the most prescribed inhaled antibiotic in the USA, whereas Colistin was prescribed most in the UK (online supplemental material section 10).

### Robustness test

In both countries lung function decline was less steep in the population born after 1997 compared with the whole study population. The estimated gap in absolute level of lung function was comparable to the estimates derived from the whole population (online supplemental material section 12).

## Discussion

We assessed longitudinal lung function trajectories in comparable populations of children in the USA and UK using data from national registries and found that lung function was better in US children over the entire age range from 6 up to age 18 years. For example, based on the best fitting longitudinal model, females in the USA were estimated to have approximately 3.60 (95% CI 2.40 to 4.80) and 5.80 (95% CI 3.88 to 7.62) percentage points higher %FEV_1_ than females in the UK at age 6 and 17, respectively. The gap was slightly smaller for males. Children in the USA with CF may have a slower rate of decline in lung function compared with UK children, but the strength of evidence for this was sensitive to modelling choices and was less clear in more recent cohorts born after 1997.

### Strengths and limitations

A major strength of this study is the use of high quality, longitudinal data with broad national coverage in each country and the application of statistical methods suitable for longitudinal data analysis which enabled us to compare population level mean lung function trajectories across childhood. We fitted a range of models to reduce the possibility that estimated differences in lung function were due to suboptimal model fit in the populations. Our main results were consistent across models.

A further strength is the reduction of differences in case-mix between the two populations through the restriction to the F508del homozygous population and the adjustment for important covariates. We also reduced the influence of survivor bias by only considering the paediatric populations. However, this does mean that our results may not be generalisable to the whole CF population and further studies are needed to understand whether the differences we found also exist for different genotypes.

The previous study by Goss *et al* 9 accounted for differences in data collection methods between registries by merging the US and UK datasets into a single data set and matching a single US clinic visit to a UK annual visit based on month of calendar year. In this study we did not try to harmonise the datasets but rather used identical inclusion/exclusion criteria and analysis methods. By including all available data points, we can more precisely estimate trends in lung function and rates of decline, but this may lead to bias due to seasonality or time between clinic visits (online supplemental material section 1). However, we do not expect this to have a major impact on our results as seasonality has been shown to have a negligible effect on lung function.^15 24^ The difference in the number of data points between the two populations is also unlikely to have significantly impacted our results based on preliminary findings from a parallel investigation of different analysis strategies applied to the US CFFPR. The potential impacts of these strategies on estimating lung function decline have been reported in abstract form from a Cystic Fibrosis Foundation workgroup.^25^ The findings show that lung function trajectory estimates were similar if using all available encounter-level %FEV_1_ data, median quarterly data or median annual data. It has been noted that in the UK CF population, the average best measured %FEV_1_ per year is significantly higher (approximately 4 percentage points) than the annual review %FEV_1_ that we used in this study.^2 26^ Best FEV_1_ has only been collected in the UK CF Registry since 2012 and therefore we could not conduct a full comparison of this measure between the two countries. However, a cross-sectional description of annual vs best %FEV_1_ in the UK and average-per-year vs best %FEV_1_ in the USA, showed similar differences in the two countries and indicated that an analysis using best %FEV_1_ would likely return similar results to our findings (see online supplemental material section 13).

Although we adjusted for a number of baseline covariates, a limitation remains the potential difference in casemix, especially due to socioeconomic factors. These have been shown to impact outcomes in CF^14 27 28^ but due to differences in measuring socioeconomic status between countries and differences in data collection between registries, it was not possible to adjust for these. It has previously been suggested that the US CFFPR may not capture the full spectrum of socioeconomic conditions as the UK CF Registry does. A sensitivity analysis of this assumption in relation to the study by Goss *et al*, however did not alter their results significantly and is therefore not likely to have a major impact on our findings.^29^ A related open question is whether individuals not captured in the registries differ significantly from those who are. If this is the case, then any results from analyses of the registry population may not be generalisable to the population as a whole. Therefore, when interpreting the findings from our study, we need to be mindful that about 16% of the US CF population is not captured by the CFFPR and in the UK it is only since 2012 that 99% have been captured.

A further limitation is the generalisability of these results to the CF population in the era of new cystic fibrosis transmembrane conductance regulator (CFTR) modulators. In future studies, it will be important to assess whether the observed differences have widened with the earlier introduction of Orkambi (lumacavtor/ivacavtor) in the USA compared with the UK and whether we can expect the difference to decrease in the next generation when both countries will have access to CFTR modulators, including the triple combination therapy, for almost the whole population. However, the fast-paced development and the variation in introduction and eligibility criteria for CFTR modulator therapies across different countries pose additional difficulties for conducting cross-country comparison analyses of registry data.

### Comparison with previous studies

Our overarching finding, that the paediatric homozygous F508del population in the USA had higher lung function than this population in the UK, corroborates the results from the cross-sectional study by Goss *et al*. However, the longitudinal nature of our study enabled us to compare the population-level mean lung function trajectories throughout childhood. We showed that the gap between the two populations was sustained and may even be increasing during childhood and adolescence. These findings are in contrast to Goss *et al* who had previously found that cross-sectionally the gap in lung function between the USA and UK appeared to be decreasing in adolescence and disappeared at age 30. Their findings are likely due to cohort effects and could be explained by our result that the gap between the two countries may be widening with calendar time.

### Implications for clinical practice

The sustained gap in lung function between the USA and UK which may be increasing with age and calendar time is a major concern for the UK CF community. As suboptimal nutritional status early in life is associated with lower pulmonary function,^30^ we compared anthropomorphic measures between the two study populations. Children in our UK study population had on average better early nutritional status than children in the US study population. If anything, this would portend, if all other things were equal, that lung function in the UK would be better than in the USA. In other words, the factors contributing to the difference in lung function between the two populations may be responsible for an even greater difference than just the difference reported in our study.

Additional factors such as differential use of medications between the countries may impact lung function.^31 32^ We found big differences in the proportion of children receiving rhDNase and hypertonic saline with much more aggressive treatment at younger ages in the USA compared with the UK. In the pivotal study, once daily rhDNase reduced pulmonary exacerbations by 28% and improved FEV_1_ by 5.8% compared with placebo^32^ but a recent study in the UK found that rhDNase may only improve lung function in individuals with reduced FEV_1_ at baseline.^33^ In both studies, however, children under 5 years of age were excluded and there remains a paucity of data on the use of rhDNase in preschool children. Although overall levels of prescribed inhaled antibiotics were similar between the countries, US children were almost entirely treated with Tobramycin. Whereas in the UK, colistin was used almost exclusively, especially in children under the age of 6. These results raise the question whether the earlier prescription of mucoactive agents in the USA led to improved lung function at age 6, or whether differences between the US and UK in early antibiotic use, both nebulised and oral,^34^ may have an impact on lung function. Further work will be needed to investigate this and other potential reasons for the observed differences. This will include investigating whether there are differences in lung function between the healthy populations in the USA and the UK. We will also need to understand the generalisability of these findings to the whole CF population and the implications for long term outcomes including survival or time to lung transplant.

## Conclusion

Using patient registry data to conduct international comparisons of health among people with CF is challenging due to variation in data collection methods but may highlight differences in healthcare and health outcomes between countries. In our comparative longitudinal analysis of children with CF, we found significant differences in lung function between the USA and UK. Children in the USA had higher lung function at age six which was sustained throughout childhood and adolescence. The rate of decline in lung function with age may also be slower in the USA. Further work will need to investigate possible reasons for the gap in lung function between the USA and the UK at age 6 including a more detailed analysis of treatment patterns.

## Data Availability

Data may be obtained from a third party and are not publicly available. The data used in this study are from the US CF Foundation Patient Registry and the UK CF Registry. The data are available from the respective organisations on reasonable request.
