# Supplementary material for: Lung function in children with cystic fibrosis in the USA and UK: a comparative longitudinal analysis of national registry data
Source: Thorax. 2021 May 11;77(2):136–42. doi: 10.1136/thoraxjnl-2021-216849 (PMC8581063; doi:10.1136/thoraxjnl-2021-216849)
Supplement: Supplementary data [file thoraxjnl-2021-216849supp001.pdf]

Lung function in children with cystic fibrosis in the US and UK: A comparative longitudinal study

Supplementary Material

Daniela K. Schlüter Joshua S. Ostrenga Siobhán B. Carr Aliza K. Fink
Albert Faro Rhonda D. Szczesniak Ruth H. Keogh Susan C. Charman
Bruce C. Marshall Christopher H. Goss David Taylor-Robinson

Schlüter DK, *et al. Thorax* 2022; 77:136–142. doi: 10.1136/thoraxjnl-2021-216849

1. Comparison of healthcare practices and CF registries

The CFFPR and the UK CF Registry

We utilised the US CFFPR and the UK CF Registry. The CFFPR was established in the 1960s to collect information on patient demographics and survival. With advances in technology and improvements in data collection it has been maintained and updated since 1986 and now collects data on individuals treated at one of over 120 accredited care centres in the CF Foundation Care Centre Network. The current CFFPR includes data from 1986 onwards on over 50,000 patients and is estimated to capture about 84% of the current US CF population. Since 2003, CF care centres have been encouraged to enter information from all clinical encounters at CF Foundation Care Centre Network facilities (Knapp et al Ann Am Thorac Soc. 2016;13(7):1173-1179). Current CFF guidelines are for patients to be seen quarterly for routine care (https://www.cff.org/Care/Clinical-Care-Guidelines). In the UK, children with CF receive care in one of 28 specialist paediatric CF centres. The recommendation is for care teams to submit annual encounter data to the Registry from a clinic visit approximately 12 months after the previous entry and when the patient is clinically stable.Records date back to the 1990s and are estimated to capture approximately 80% of the UK CF population from 2002 onwards and 99% since 2012 (See Talor-Robinson et al Int J Epidemiol. 2018;47(1):9-10e for more details). The annual review visits include evaluation of clinical status, lung function, and microbiology of respiratory tract secretions. Since 2012 CF centres are also encouraged to enter the best FEV1 measurement since last annual review.

Data collection

The US CFFPR collects data at every encounter whereas the UK CF Registry captures data from annual review visits. The visist are relatively uniformly distributed across the year in the US. In the UK, there is a slight tendancy for the annual review visits to happen later in the year (Figure 1).

Schlüter DK, *et al. Thorax* 2022; 77:136–142. doi: 10.1136/thoraxjnl-2021-216849

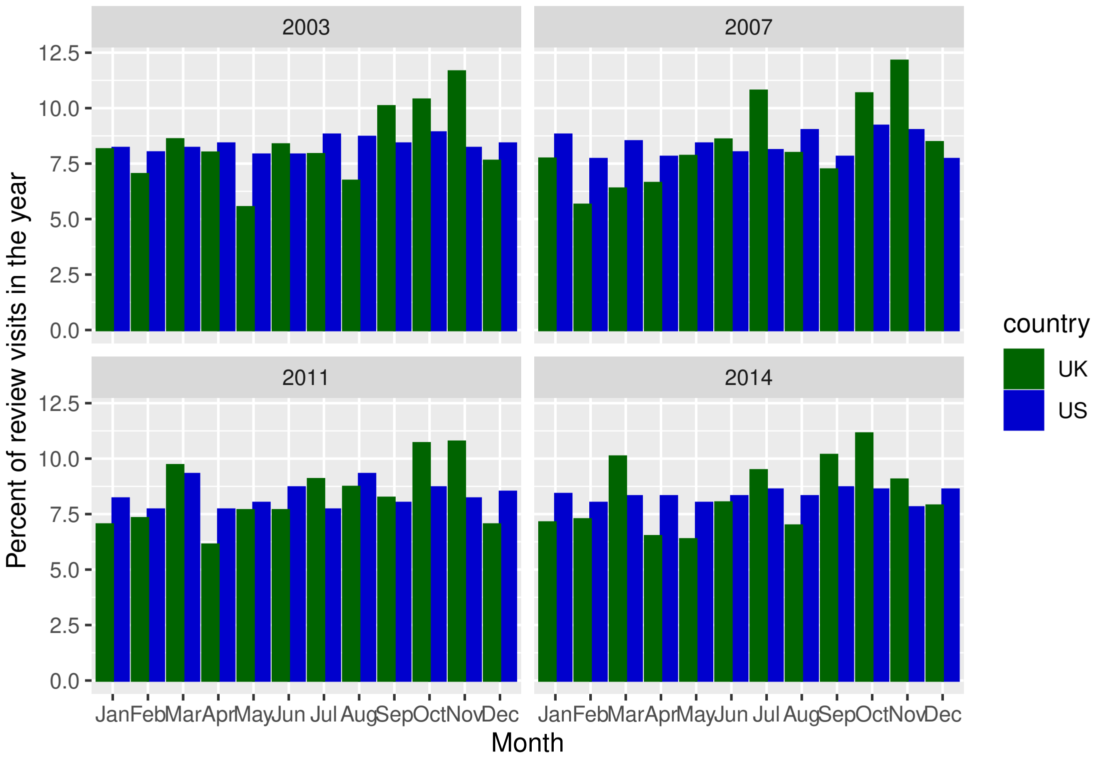

Figure 1: Comparison of seasonality of recorded clinic visits in the study population for selected years.

Diagnosis

We found differences between the US and the UK with regrad to the proprotion of individuals diagnosed by newborn screening. This difference is likely due to newborn screening being introduced at different times in the two countries. In the US universal newborn screening was introduced in 2009. In the UK regional screening programs first started in the 1980s and universal screening was introduced in 2007.

Introduction and recording of treatments

There are differences in the introduction of treatments into routine care between the US and UK. In addition treatments may not have been recorded in the registries from the time at which they were introduced which needs to be taken into account when reporting on treatment use.

Schlüter DK, *et al. Thorax* 2022; 77:136–142. doi: 10.1136/thoraxjnl-2021-216849

Table 1: Years in which treatments were introduced and first recorded in the CF registries in the US and the UK.

	US		UK	
	introduced	collected in registry	introduced	collected in registry
rhDNase	1993[a]	1995	1994[a]	1996
Hypertonic Saline	2006[b]	2005	2006[b]	1996
Tobramycin (inhaled)	1997[a]	1997	1999[a]	1996
Colistin (inhaled)	N/A[c]	2005	1981[d]	1996
Aztreonam (inhaled)	2010[a]	2005	2012[a]	2012

[a] FDA/EMA approval

[b] Year of evidence publication (Elkins MR et al.N Engl J Med 2006; 354:229-240 doi: 10.1056/NEJMoa043900)

[c] never been approved by the FDA for CF or endorsed in practice guidelines

[d] Year of evidence publication (Hodson ME et al. Lancet.1981 Nov 21;2(8256):1137-9. doi: 10.1016/s0140-6736(81)90588-2.) which lead to slow adoption as standard practice in the UK

Schlüter DK, *et al. Thorax* 2022; 77:136–142. doi: 10.1136/thoraxjnl-2021-216849

2. Exploratory graphical data analysis

We initially explored scatterplots of % predicted FEV1 by age in the US and the UK to examine changes in the population level mean trend over time (see Figure 2).

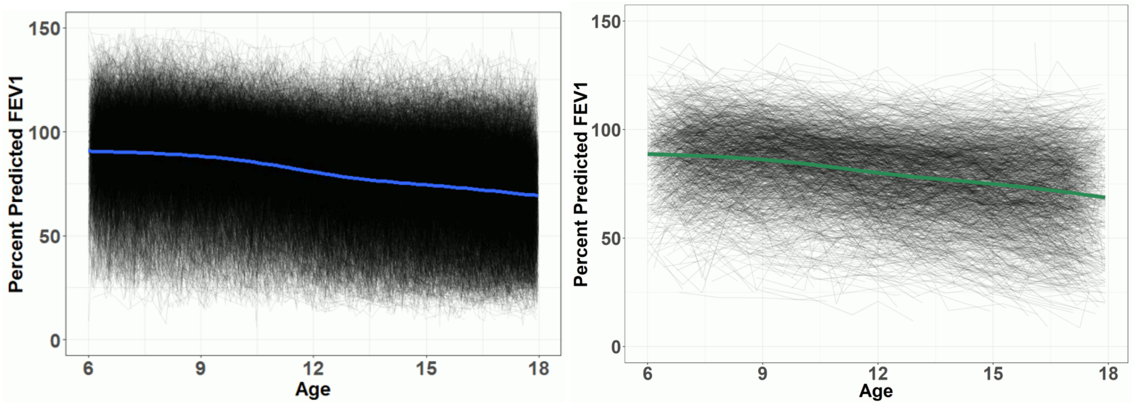

Figure 2: % predicted FEV1 against age in the US population (left panel) and UK population (right panel). The black lines are individual trajectories, the blue/green line is a smoother representing the population mean at each age.

We also used scatterplot smoothing to examine the relationship of each covariate with FEV1% (Figures 3-5).

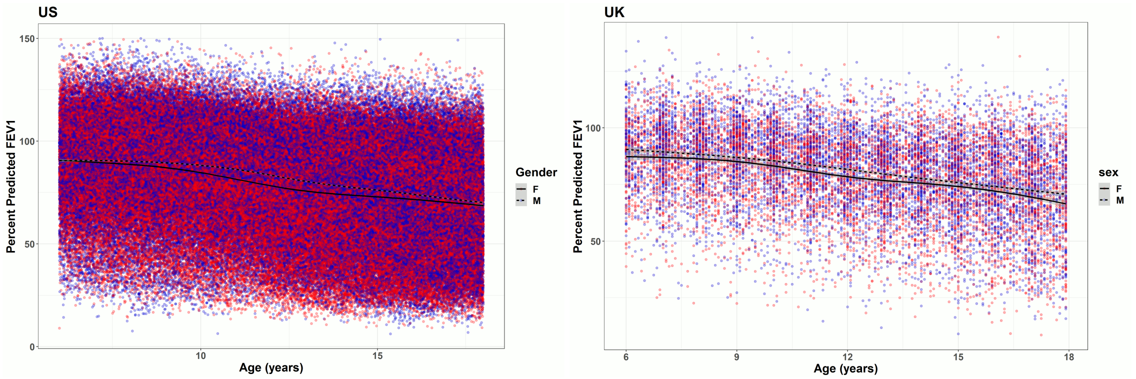

Figure 3: % predicted FEV1 against age in the US population (left panel) and UK population (right panel) coloured by sex. The black lines are smoothers by sex.

Schlüter DK, *et al. Thorax* 2022; 77:136–142. doi: 10.1136/thoraxjnl-2021-216849

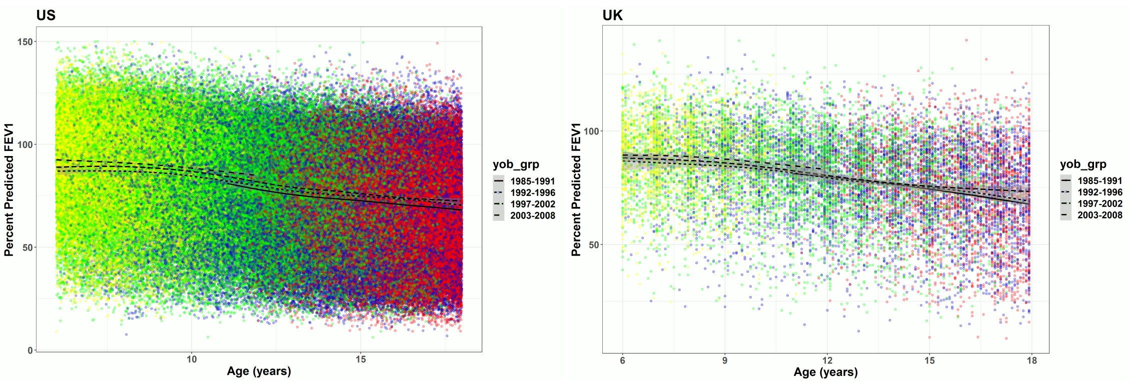

Figure 4: % predicted FEV1 against age in the US population (left panel) and UK population (right panel) coloured by year of birth. The black lines are smoothers by birth cohort.

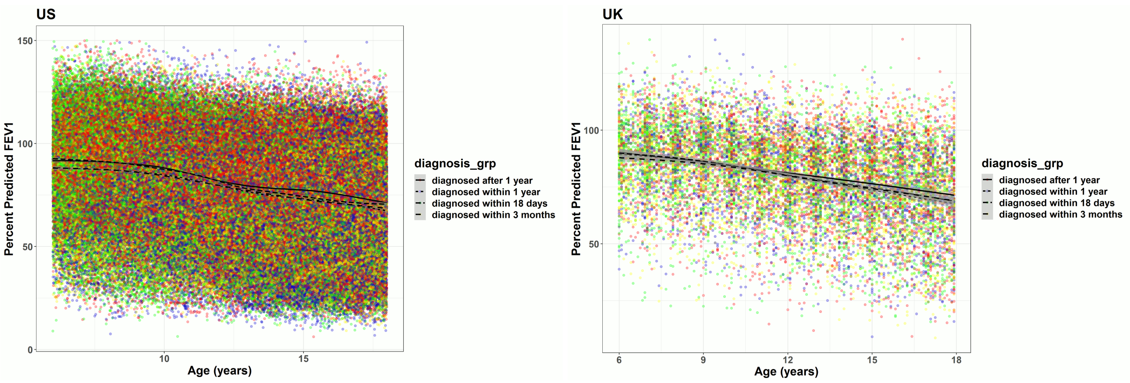

Figure 5: % predicted FEV1 against age in the US population (left panel) and UK population (right panel) coloured by age at diagnosis. The black lines are smoothers by age at diagnosis grouped into *diagnosed between birth and 18 days, diagnosed between 19 days and 3 months, diagnosed between 3 months and 1 year and diagnosed after one year.*

In figures 6 and 7 we highlight three randomly selected individual trajectories in the US and UK, respectively.

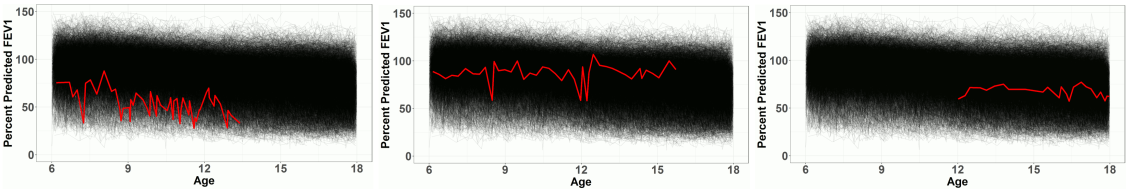

Figure 6: % predicted FEV1 against age in the US population with 3 randomly selected individuals higlighted in red.

Schlüter DK, *et al. Thorax* 2022; 77:136–142. doi: 10.1136/thoraxjnl-2021-216849

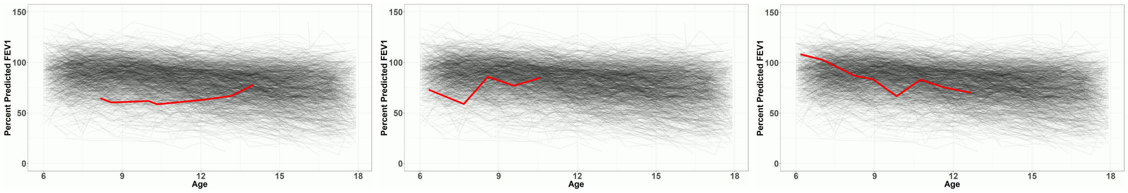

Figure 7: % predicted FEV1 against age in the UK population with 3 randomly selected individuals higlighted in red.

To identify resonable models for the correlation between repeated measurements, we plotted the variograms of the residuals after fitting a general additive model as a mean smoother (shown in Figure 2). Figure 8 shows the variograms for the US and the UK data.

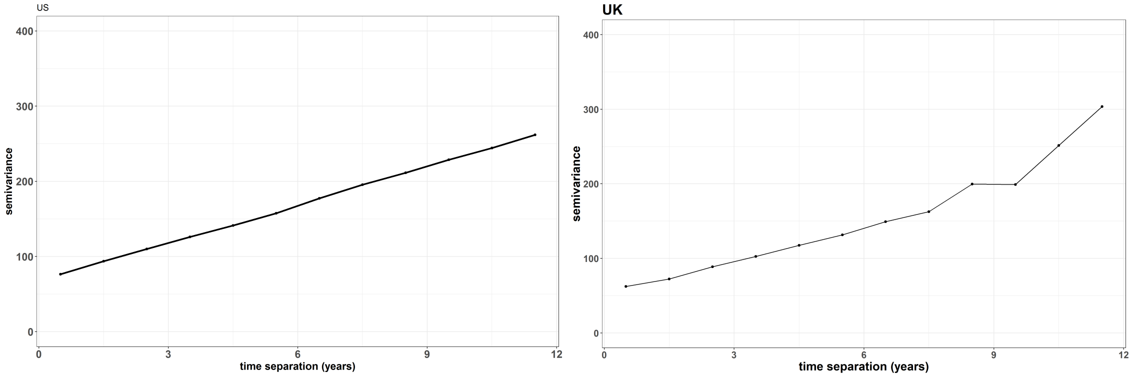

Figure 8: Variogram of the residuals after fitting a general additive model to the data in the US (left panel) and UK (right panel).

Schlüter DK, *et al. Thorax* 2022; 77:136–142. doi: 10.1136/thoraxjnl-2021-216849

3. Model specifications

In the UK, previously published approaches modelled population-level mean lung function as linear functions of age (Taylor-Robinson et al Lancet Respir Med. 2013;1(2):121-128; Qvist et al J Cyst Fibros Off J Eur Cyst Fibros Soc. 2019;18(3):390-395), whereas in the US, cubic functions and semi-parametric approaches have been utilised to capture non-linear behaviours (Szczesniak et al Ann Epidemiol. 2013;23(12):771-777). Therefore, we fitted a series of models of different complexity to the US and UK study populations. The models fitted in this study are of the form

$$y_{ij} = x_i^{'}\beta + f(t_{ij}) + Z_{ij} \quad (1)$$

where

y_{ij} is % of predicted FEV_1 in individual i at observation j

x_i is the vector of baseline covariates for individual i (sex, year of birth, age at diagnosis)

t_{ij} is time of observation j in individual i measured as time since age 6.

We consider different functional forms for $f(t)$:

1. linear function : $f(t) = \beta_1 t$
2. quadratic function : $f(t) = \beta_1 t + \beta_2 t^2$
3. cubic function : $f(t) = \beta_1 t + \beta_2 t^2 + \beta_3 t^3$
4. cubic natural spline model with K=1,2,5 and 11 knots : $f(t) = \sum_{k=1}^{K+1} \beta_k B_k(t)$ where $B_k(t)$ are a set of basis functions. We use the function ns() in the R-package splines to generate the B-spline basis matrix for a natural cubic spline.

We further consider different assumptions for the correlation of errors Z_{ij} within an individual:

1. random intercept model:
$$Z_{ij} = U_i + \epsilon_{ij}$$
where $U_i \sim N(0, \sigma_V)$ is the random intercept and $\epsilon_{ij} \sim N(0, \sigma)$ is the measurement error
2. random intercept and slope model :
$$Z_{ij} = U_i + V_i t_{ij} + \epsilon_{ij}$$
where U_i and V_i are the random intercept and slope, respectively, with $(U_i, V_i) \sim BVN(0, \Sigma_{UV})$ and $\epsilon_{ij} \sim N(0, \sigma)$ is the measurement error
3. random intercept model with serial correlation:
$$Z_{ij} = U_i + W(t_{ij}) + \epsilon_{ij}$$
where the U_i, and ϵ_{ij} are as in 1. above. The $W(t_{ij})$ are sampled from independent copies of a stationary Gaussian process with mean zero, variance σ_W and correlation funcion $\rho(u)$; we consider here
   (a) the exponential correlation function :
   $\rho(u) = exp(-\phi u) = exp(-\phi|t_{ij} - t_{im}|)$ for observations j and m
   (b) the linear correlation funtion bounded in u:
   $\rho(u) = (1 - u/\phi)I(u < \phi) = (1 - |t_{ij} - t_{im}|/\phi)I(|t_{ij} - t_{im}| < \phi)$ for observations j and m.
4. random intercept and slope model with serial correlation:
$$Z_{ij} = U_i + V_i t_{ij} + W(t_{ij}) + \epsilon_{ij}$$
where the U_i, V_i, $W(t_{ij})$ and ϵ_{ij} are as above. Here we only consider the exponential correlation model.

4. Derivation of the study population

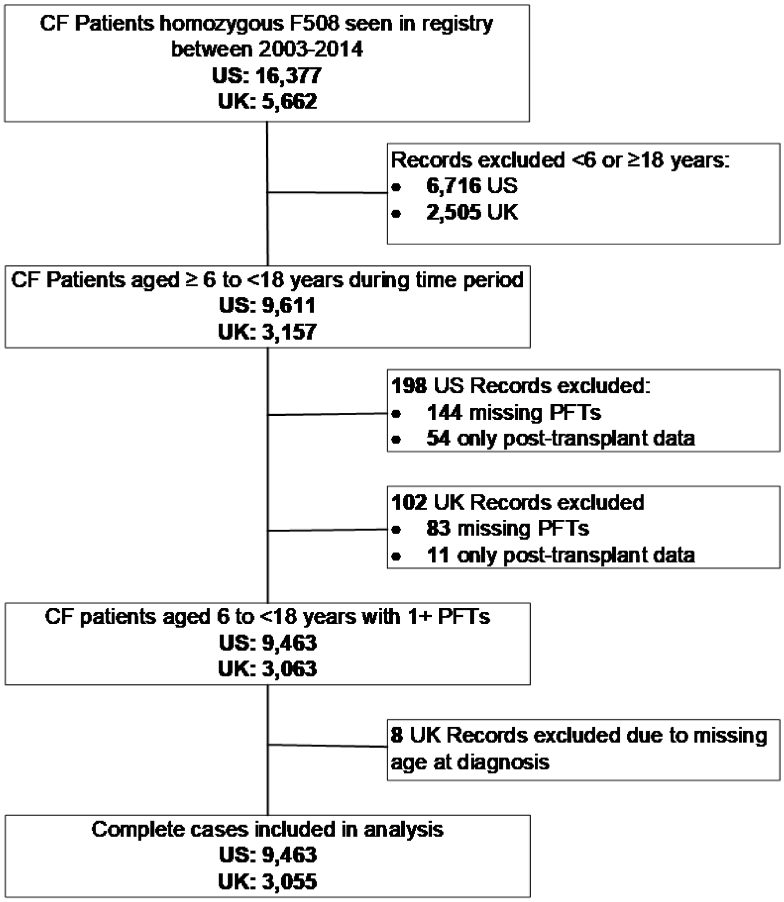

Figure 9: Derivation of the study population. PFT: pulmonary function test.

Schlüter DK, *et al. Thorax* 2022; 77:136–142. doi: 10.1136/thoraxjnl-2021-216849

5. Distribution of the year of birth

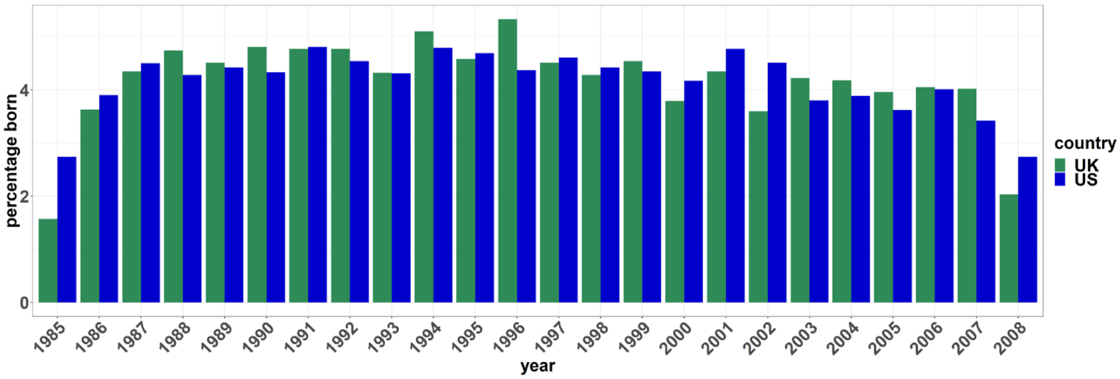

Figure 10: Distribution of the year of birth in the US and UK study populations

Schlüter DK, *et al. Thorax* 2022; 77:136–142. doi: 10.1136/thoraxjnl-2021-216849

6. Within country comparisons of models

Table 2 shows the fit statistics for the different models for the US and UK. In both countries the model that included a linear function of age with random intercept, random slope and serial correlation with exponential correlation function gave the best fit. This was closely followed by the model that included age using a spline with 5 knots, random intercept and exponential correlation function. Figure 11 shows that the estimated population level mean lung function trajectories for all the different models are very similar. Figure 12 shows the estimated population-level mean rates of decline based on the different models.
Table 3 gives the covariate effect estimated from all the different models.

Table 2: Fit statistics for fitted models. 'RI': random intercept; 'RS': random slope; 'SC': serial correlation 'exp':exponential correlation function; 'lin': linear correlation function.

	US			UK		
functional form for age	-2 log likelihood	AIC	BIC	-2 log likelihood	AIC	BIC
linear function with						
RI	2070788.74	2070802.74	2070876.27	117723.9	117737.9	117791.13
RI + RS	2022109.78	2022127.78	2022222.31	116220.81	116238.81	116307.24
RI + SC exp	1996529.54	1996547.54	1996642.08	116025.41	116043.41	116111.84
RI + SC lin	1996709.86	1996727.85	1996822.39	116050.09	116068.09	116136.52
RI + RS + SC exp	1995752.42	1995774.41	1995889.96	115850.24	115872.24	115955.88
quadratic function with						
RI	2069722.88	2069738.88	2069822.91	117656.93	117672.93	117733.76
RI + SC exp	1996231.7	1996251.71	1996356.74	115978.84	115998.84	116074.88
RI + SC lin	1996412.32	1996432.32	1996537.36	115990.21	116010.21	116086.24
cubic function with						
RI	2068827.16	2068845.17	2068939.7	117621.41	117639.41	117707.84
RI + SC exp	1996034.9	1996056.89	1996172.43	115946.49	115968.49	116052.13
RI + SC lin	1996208.34	1996230.34	1996345.88	115956.01	115978.01	116061.65
spline with 1 knot and						
RI	2069827.6	2069843.5	2069927.6	117663.34	117679.34	117740.17
RI + SC exp	1996284.82	1996304.82	1996409.86	115985.48	116005.48	116081.51
RI + SC lin	1996468.22	1996488.21	1996593.25	115996.91	116016.91	116092.94
spline with 2 knots and						
RI	2068787.6	2068805.7	2068900.2	117623.49	117641.49	117709.92
RI + SC exp	1996021.76	1996043.75	1996159.3	115948.64	115970.64	116054.28
RI + SC lin	1996198.54	1996220.55	1996336.09	117619.15	117641.15	117724.78
spline with 5 knots and						
RI	2068244.6	2068268.7	2068394.7	117599.33	117623.33	117714.57
RI + SC exp	1995801.38	1995829.39	1995976.44	115923.38	115951.38	116057.83
RI + SC lin	1995958.98	1995986.98	1996134.03	117595.06	117623.06	117729.51
spline with 11 knots and						
RI	2068237.6	2068273.6	2068462.7	117593.45	117629.45	117766.31
RI + SC exp	1995797	1995837.01	1996047.08	115917.94	115957.94	116110.01
RI + SC lin	1996392.44	1996432.44	1996642.51	117589.22	117629.22	117781.28

Schlüter DK, *et al. Thorax* 2022; 77:136–142. doi: 10.1136/thoraxjnl-2021-216849

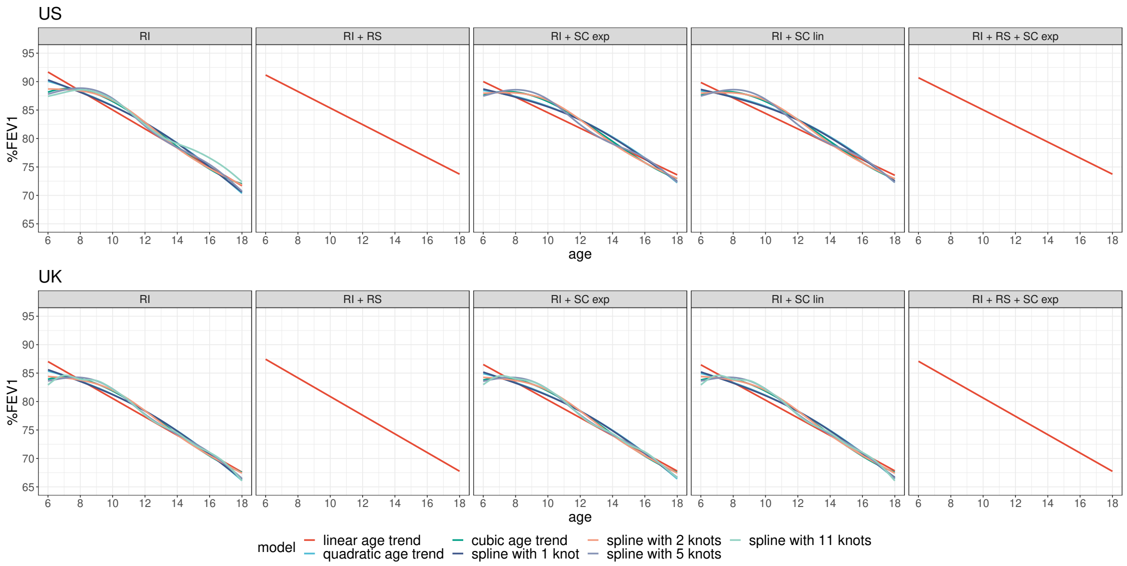

Figure 11: Estimated population average lung function trajectories at reference covariate values (females, born in 1997 and diagnosed at birth) for the different models.

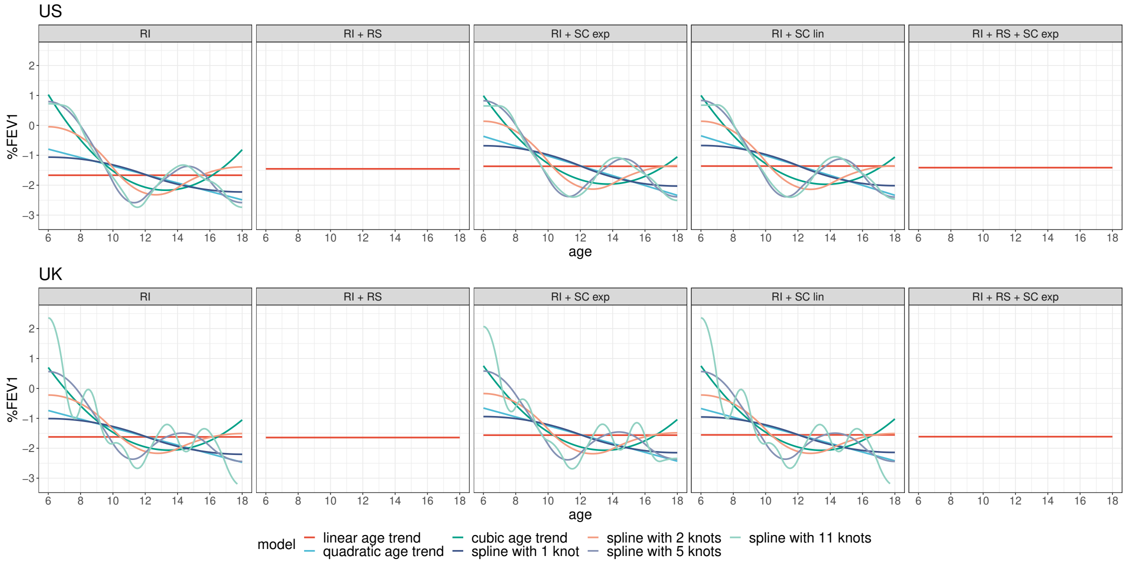

Figure 12: Estimated population average rate of lung function decline for the different models.

Schlüter DK, *et al. Thorax* 2022; 77:136–142. doi: 10.1136/thoraxjnl-2021-216849

Table 3: Covariate effect estimates (standard errors) for the different models. 'RI': random intercept; 'RS': random slope; 'SC': serial correlation 'exp':exponential correlation function; 'lin': linear correlation function.

	US			UK		
functional form for age	sex	age at diagnosis	year of birth	sex	age at diagnosis	year of birth
linear function with						
RI	1.92 (0.39)	0.59 (0.08)	0.30 (0.03)	2.68 (0.62)	0.61 (0.16)	0.27 (0.05)
RI + RS	1.78 (0.38)	0.58 (0.08)	0.38 (0.03)	2.52 (0.6)	0.6 (0.16)	0.23 (0.05)
RI + SC exp	1.79 (0.39)	0.58 (0.08)	0.42 (0.03)	2.71 (0.62)	0.62 (0.16)	0.29 (0.06)
RI + SC lin	1.75 (0.39)	0.59 (0.08)	0.42 (0.03)	2.72 (0.62)	0.62 (0.16)	0.29 (0.06)
RI + RS + SC exp	1.42 (0.37)	0.59 (0.08)	0.37 (0.03)	2.5 (0.6)	0.62 (0.16)	0.24 (0.06)
quadratic function with						
RI	1.92 (0.39)	0.58 (0.08)	0.30 (0.03)	2.68 (0.62)	0.59 (0.16)	0.28 (0.05)
RI + SC exp	1.80 (0.39)	0.56 (0.08)	0.43 (0.03)	2.71 (0.62	0.6 (0.16	0.3 (0.06
RI + SC lin	1.76 (0.39)	0.57 (0.08)	0.43 (0.03)	2.73 (0.62	0.6 (0.16	0.3 (0.06
cubic function with						
RI	1.93 (0.39)	0.57 (0.08)	0.33 (0.03)	2.69 (0.62)	0.58 (0.16)	0.29 (0.05)
RI + SC exp	1.81 (0.39)	0.57 (0.08)	0.42 (0.03)	2.72 (0.62)	0.6 (0.16)	0.3 (0.06)
RI + SC lin	1.77 (0.39)	0.57 (0.08)	0.41 (0.03)	2.74 (0.62)	0.59 (0.16)	0.3 (0.06)
spline model with 1 knot and						
RI	1.92 (0.39)	0.58 (0.08)	0.30 (0.03)	2.68 (0.62)	0.59 (0.16)	0.27 (0.05)
RI + SC exp	1.80 (0.39)	0.57 (0.08)	0.43 (0.03)	2.71 (0.62)	0.6 (0.16)	0.3 (0.06)
RI + SC lin	1.76 (0.39)	0.57 (0.08)	0.43 (0.03)	2.73 (0.62)	0.6 (0.16)	0.3 (0.06)
spline model with 2 knots and						
RI	1.93 (0.39)	0.57 (0.08)	0.33 (0.03)	2.69 (0.62)	0.59 (0.16)	0.29 (0.05)
RI + SC exp	1.81 (0.39)	0.57 (0.08)	0.41 (0.03)	2.72 (0.62)	0.6 (0.16)	0.3 (0.06)
RI + SC lin	1.77 (0.39)	0.57 (0.08)	0.41 (0.03)	2.69 (0.62)	0.59 (0.16)	0.29 (0.05)
spline model with 5 knots and						
RI	1.93 (0.39)	0.57 (0.08)	0.33 (0.03)	2.68 (0.62)	0.59 (0.16)	0.29 (0.05)
RI + SC exp	1.80 (0.39)	0.57 (0.08)	0.42 (0.03)	2.71 (0.62)	0.6 (0.16)	0.3 (0.06)
RI + SC lin	1.76 (0.39)	0.57 (0.08)	0.41 (0.03)	2.68 (0.62)	0.59 (0.16)	0.29 (0.05)
spline model with 11 knots and						
RI	1.93 (0.39)	0.57 (0.08)	0.33 (0.03)	2.68 (0.62)	0.59 (0.16)	0.29 (0.05)
RI + SC exp	1.80 (0.39)	0.57 (0.08)	0.42 (0.03)	2.71 (0.62)	0.6 (0.16)	0.3 (0.06)
RI + SC lin	1.79 (0.39)	0.57 (0.08)	0.42 (0.03)	2.68 (0.62)	0.59 (0.16)	0.29 (0.05)

Schlüter DK, *et al. Thorax* 2022; 77:136–142. doi: 10.1136/thoraxjnl-2021-216849

7. Estimated difference in lung function between the US and the UK for different covariate values

The estimated difference in lung function between the US and UK CF populations is dependent on covariate values. In tables 4 and 5 we report the difference at selected ages and for selected combinations of covariate values based on the model that included a linear function of age and the model that included age using a spline with 5 knots, respectively.

Table 4: Estimated difference in lung function between the US and UK populations based on selected sets of covariate values and the model that included a linear function of age with random interept, random slope and exponential correlation function.

	females			males		
year of birth	age 6	age 12	age 17	age 6	age 12	age 17
diagnosed at birth						
1990	2.71 (1.05, 4.37)	3.91 (2.52, 5.3)	4.91 (3.47, 6.34)	1.63 (0, 3.26)	2.83 (1.47, 4.18)	3.83 (2.43, 5.23)
1997	3.6 (2.4, 4.8)	4.8 (3.73, 5.88)	5.8 (4.49, 7.11)	2.52 (1.36, 3.69)	3.72 (2.69, 4.76)	4.72 (3.44, 6)
2004	4.5 (3.21, 5.79)	5.7 (4.32, 7.07)	6.69 (4.99, 8.4)	3.42 (2.15, 4.68)	4.62 (3.26, 5.97)	5.62 (3.93, 7.3)
diagnosed at age 1						
1990	2.67 (1.07, 4.28)	3.87 (2.55, 5.19)	4.87 (3.51, 6.23)	1.59 (0.02, 3.16)	2.79 (1.51, 4.07)	3.79 (2.46, 5.12)
1997	3.57 (2.42, 4.72)	4.77 (3.75, 5.78)	5.76 (4.51, 7.02)	2.49 (1.37, 3.6)	3.69 (2.71, 4.66)	4.68 (3.46, 5.91)
2004	4.46 (3.19, 5.73)	5.66 (4.31, 7.01)	6.66 (4.98, 8.34)	3.38 (2.14, 4.62)	4.58 (3.25, 5.91)	5.58 (3.91, 7.24)

Table 5: Estimated difference in lung function between the US and UK populations based on selected sets of covariate values and the model that included age using a spline with 5 knots and random interept and exponential correlation function.

	females			males		
year of birth	age 6	age 12	age 17	age 6	age 12	age 17
diagnosed at birth						
1990	3.02 (1.12, 4.92)	3.93 (2.43, 5.43)	4.83 (3.45, 6.22)	2.13 (0.26, 4)	3.04 (1.57, 4.5)	3.94 (2.59, 5.29)
1997	3.87 (2.36, 5.37)	4.77 (3.54, 6)	5.68 (4.4, 6.96)	2.97 (1.5, 4.45)	3.88 (2.69, 5.08)	4.79 (3.54, 6.03)
2004	4.71 (3.12, 6.3)	5.62 (4.07, 7.17)	6.52 (4.79, 8.26)	3.82 (2.25, 5.39)	4.73 (3.2, 6.26)	5.63 (3.92, 7.35)
diagnosed at age 1						
1990	2.98 (1.13, 4.84)	3.89 (2.46, 5.33)	4.8 (3.48, 6.11)	2.09 (0.27, 3.92)	3 (1.6, 4.4)	3.91 (2.63, 5.18)
1997	3.83 (2.37, 5.29)	4.74 (3.56, 5.91)	5.64 (4.42, 6.87)	2.94 (1.51, 4.37)	3.85 (2.71, 4.99)	4.75 (3.56, 5.95)
2004	4.68 (3.1, 6.25)	5.58 (4.06, 7.11)	6.49 (4.78, 8.2)	3.79 (2.23, 5.34)	4.69 (3.19, 6.2)	5.6 (3.91, 7.29)

Schlüter DK, *et al. Thorax* 2022; 77:136–142. doi: 10.1136/thoraxjnl-2021-216849

8. Estimated rates of decline based on the model that included age using a spline with 5 knots

The estimated rate of lung function decline based on the model that included age using a spline with 5 knots changes with age. This is illustrated in Figure 1 in the main paper. Table 6 gives the estimates for the rate of decline at a selected set of ages for females, born in 1997 and diagnosed at birth. It also gives the differences between the estimated rates of decline in the US and the UK.

Table 6: Estimated rates of decline (95% confidence intervals) in the US and UK populations at selected ages based on the spline model with 5 knots, random intercept and exponential correlation function. We show both results for the whole population and for the population born after 1997. We also give the differences in the rates of decline etween the US and the UK.

	Whole population			Population born after 1997		
Age	US	UK	difference	US	UK	difference
6	0.82 (0.56, 1.08)	0.58 (-0.15, 1.31)	0.24 (-0.53, 1.02)	0.85 (0.57, 1.13)	0.73 (-0.1, 1.55)	0.12 (-0.75, 0.99)
7	0.62 (0.4, 0.84)	0.38 (-0.2, 0.96)	0.24 (-0.38, 0.86)	0.64 (0.42, 0.86)	0.47 (-0.16, 1.11)	0.16 (-0.51, 0.84)
8	0.01 (-0.29, 0.31)	-0.21 (-0.51, 0.09)	0.23 (-0.2, 0.65)	0.01 (-0.13, 0.15)	-0.29 (-0.6, 0.02)	0.3 (-0.04, 0.64)
9	-0.83 (-1.22, -0.45)	-1.02 (-1.48, -0.57)	0.19 (-0.41, 0.78)	-0.88 (-1.08, -0.67)	-1.2 (-1.74, -0.67)	0.32 (-0.25, 0.9)
10	-1.76 (-1.89, -1.64)	-1.87 (-2.16, -1.57)	0.11 (-0.21, 0.43)	-1.85 (-2.01, -1.69)	-1.89 (-2.22, -1.56)	0.04 (-0.33, 0.41)
11	-2.35 (-2.73, -1.97)	-2.37 (-2.82, -1.93)	0.02 (-0.56, 0.61)	-2.45 (-2.68, -2.22)	-2.19 (-2.78, -1.61)	-0.25 (-0.88, 0.38)
12	-2.18 (-2.48, -1.88)	-2.16 (-2.45, -1.87)	-0.02 (-0.43, 0.4)	-2.21 (-2.4, -2.03)	-1.95 (-2.33, -1.57)	-0.27 (-0.69, 0.16)
13	-1.67 (-1.85, -1.49)	-1.71 (-2.15, -1.27)	0.04 (-0.43, 0.51)	-1.65 (-1.92, -1.38)	-1.58 (-2.25, -0.91)	-0.07 (-0.79, 0.66)
14	-1.24 (-1.37, -1.12)	-1.48 (-1.76, -1.19)	0.24 (-0.08, 0.55)	-1.25 (-1.49, -1.01)	-1.52 (-2.01, -1.02)	0.26 (-0.29, 0.81)
15	-1.15 (-1.32, -0.98)	-1.51 (-1.95, -1.08)	0.37 (-0.1, 0.84)	-1.16 (-1.51, -0.8)	-1.42 (-2.29, -0.55)	0.26 (-0.68, 1.2)
16	-1.62 (-1.74, -1.5)	-1.86 (-2.15, -1.58)	0.24 (-0.07, 0.55)	-1.5 (-1.87, -1.13)	-0.95 (-1.76, -0.15)	-0.54 (-1.43, 0.35)
17	-2.2 (-2.39, -2)	-2.24 (-2.81, -1.67)	0.04 (-0.56, 0.65)	-1.91 (-2.69, -1.14)	-0.47 (-2.49, 1.56)	-1.44 (-3.61, 0.72)

Schlüter DK, *et al. Thorax* 2022; 77:136–142. doi: 10.1136/thoraxjnl-2021-216849

9. Early Growth from first recording in Registry to <18 years

In tables 7, 8 and 9 we present descriptive indicators of growth and nutrition cross-sectionally from birth to age 18 for the two study populations. We summarised height, weight and BMI by their median, 25th and 75th percentile at each year of age for all individuals in the study population for whom data were available at that age. This may include records collected prior to 2003 but observations remained censored at transplant.

Table 7: Weight

		US			UK			
birth cohort	age	N	kg	z-score (CDC)	N	kg	z-score (CDC)	z-score (UK WHO)
1985 - 1996	[0-1)	2780	6.1 [5.1- 7.1]	-1.6 [-2.3- -0.8]	6	8.9 [6.6-10.1]	-0.8 [-0.9-0.9]	-0.8 [-1.7-0.9]
	[1-2)	3080	10.3 [9.3- 11.3]	-0.9 [-1.7- -0.1]	21	10.7 [8.8-11.4]	-0.3 [-1.5-0.7]	0 [-0.9-1]
	[2-3)	3452	12.6 [11.6- 13.7]	-0.4 [-1.2- 0.2]	50	12.6 [11.8-14.3]	-0.4 [-1.1-0.5]	-0.2 [-0.9-0.5]
	[3-4)	3638	14.5 [13.4- 15.7]	-0.3 [-1.0- 0.3]	105	15 [13.9-16.4]	0.1 [-0.5-0.7]	-0.1 [-0.6-0.5]
	[4-5)	3741	16.4 [15.1- 17.8]	-0.4 [-1.0- 0.3]	188	16.7 [15.3-18.4]	-0.1 [-0.7-0.5]	-0.4 [-1-0.3]
	[5-6)	3954	18.2 [16.8- 19.9]	-0.4 [-1.1- 0.2]	311	18.9 [17.2-20.6]	-0.2 [-0.7-0.5]	-0.3 [-0.9-0.3]
	[6-7)	4116	20.3 [18.7- 22.1]	-0.5 [-1.1- 0.1]	431	20.7 [19-22.7]	-0.3 [-0.8-0.3]	-0.4 [-1-0.3]
	[7-8)	4249	22.6 [20.6- 24.7]	-0.5 [-1.1- 0.1]	563	23.5 [21.4-25.8]	-0.2 [-0.8-0.4]	-0.3 [-0.9-0.4]
	[8-9)	4308	25.0 [22.8- 27.5]	-0.5 [-1.1- 0.1]	694	25.7 [23.3-28.6]	-0.3 [-0.9-0.3]	-0.4 [-1-0.3]
	[9-10)	4373	27.6 [25.1- 30.6]	-0.6 [-1.2- 0.0]	766	28.6 [25.7-32.2]	-0.3 [-1-0.3]	-0.3 [-1.1-0.4]
	[10-11)	4420	30.6 [27.6- 34.0]	-0.7 [-1.3- -0.1]	885	31.4 [28.2-35.7]	-0.4 [-1.1-0.2]	-0.4 [-1-0.3]
	[11-12)	4469	33.9 [30.3- 38.2]	-0.7 [-1.4- -0.1]	1002	35 [30.9-40.2]	-0.5 [-1.2-0.2]	-0.4 [-1.1-0.4]
	[12-13)	4488	37.9 [33.6- 43.2]	-0.7 [-1.4- 0.0]	1098	39 [34.3-45]	-0.5 [-1.2-0.2]	-0.4 [-1.1-0.5]
	[13-14)	4522	42.8 [37.8- 48.8]	-0.6 [-1.4- 0.1]	1172	43.2 [38-49.9]	-0.5 [-1.3-0.2]	-0.5 [-1.2-0.3]
	[14-15)	4518	47.8 [42.3- 53.5]	-0.5 [-1.2- 0.2]	1285	48 [43-54.3]	-0.4 [-1.2-0.2]	-0.5 [-1.2-0.2]
	[15-16)	4526	51.7 [46.3- 57.7]	-0.4 [-1.2- 0.2]	1333	52 [46.6-58.3]	-0.4 [-1.1-0.2]	-0.5 [-1.2-0.2]
	[16-17)	4521	54.3 [48.7- 60.7]	-0.5 [-1.2- 0.2]	1624	54.5 [49.1-61]	-0.4 [-1.1-0.2]	-0.6 [-1.3-0.1]
	[17-18)	4497	56.1 [50.1- 62.8]	-0.5 [-1.3- 0.1]	1625	55.2 [49.8-62.5]	-0.6 [-1.3-0.1]	-0.7 [-1.5-0]
1997-2008	[0-1)	3159	6.5 [5.9- 7.2]	-1.2 [-2.0- -0.5]	394	8.1 [5.6-9.3]	-0.3 [-1-0.6]	-0.5 [-1.7-0.3]
	[1-2)	3570	10.4 [9.6- 11.3]	-0.8 [-1.5- -0.1]	687	10.7 [9.6-11.9]	-0.2 [-1-0.5]	0.2 [-0.5-0.8]
	[2-3)	3789	12.8 [11.9- 13.9]	-0.3 [-1.0- 0.3]	960	13.1 [11.9-14.3]	0 [-0.7-0.7]	0.1 [-0.5-0.7]
	[3-4)	3954	14.8 [13.7- 15.9]	-0.2 [-0.8- 0.4]	996	15.1 [13.9-16.5]	0.1 [-0.5-0.7]	0 [-0.6-0.6]
	[4-5)	4077	16.6 [15.5- 18.0]	-0.2 [-0.8- 0.4]	1095	17 [15.6-18.5]	0.1 [-0.5-0.7]	-0.2 [-0.8-0.5]
	[5-6)	4159	18.7 [17.3- 20.2]	-0.2 [-0.8- 0.3]	1149	18.9 [17.4-20.6]	-0.1 [-0.6-0.5]	-0.2 [-0.8-0.4]
	[6-7)	4247	20.9 [19.2- 22.8]	-0.2 [-0.9- 0.3]	1198	21.1 [19.3-23.4]	-0.1 [-0.8-0.5]	-0.2 [-0.9-0.5]
	[7-8)	4296	23.4 [21.4- 25.6]	-0.2 [-0.9- 0.3]	1174	23.6 [21.4-26.2]	-0.1 [-0.8-0.5]	-0.2 [-0.9-0.4]
	[8-9)	4311	26.1 [23.7- 28.9]	-0.2 [-0.9- 0.3]	1225	26.4 [23.8-29.2]	-0.1 [-0.8-0.5]	-0.2 [-0.9-0.4]
	[9-10)	4319	28.9 [26.3- 32.4]	-0.3 [-0.9- 0.3]	1148	29.2 [26.3-32.7]	-0.2 [-0.8-0.4]	-0.2 [-0.8-0.5]
	[10-11)	4053	32.2 [29.1- 36.2]	-0.4 [-1.0- 0.3]	1086	32.5 [29-36.2]	-0.3 [-0.9-0.3]	-0.2 [-0.8-0.5]
	[11-12)	3732	35.8 [32.1- 40.6]	-0.4 [-1.0- 0.2]	1026	35.9 [32-40.8]	-0.3 [-1-0.3]	-0.2 [-0.8-0.5]
	[12-13)	3383	40.1 [35.7- 45.8]	-0.4 [-1.1- 0.3]	923	40.1 [35.6-45.4]	-0.4 [-1-0.3]	-0.2 [-0.9-0.5]
	[13-14)	3046	45.1 [40.0- 51.2]	-0.3 [-1.0- 0.3]	795	44.3 [39.5-51.1]	-0.4 [-1-0.3]	-0.3 [-1-0.4]
	[14-15)	2687	49.8 [44.5- 56.0]	-0.2 [-0.9- 0.4]	700	49.3 [43.8-55.7]	-0.3 [-1-0.3]	-0.3 [-1-0.4]
	[15-16)	2320	53.4 [47.8- 59.8]	-0.2 [-0.9- 0.4]	586	52.5 [47.5-59.4]	-0.3 [-0.9-0.3]	-0.4 [-1-0.3]
	[16-17)	1906	56.0 [50.2- 62.7]	-0.2 [-1.0- 0.4]	498	56.2 [50-62]	-0.3 [-1-0.3]	-0.4 [-1.2-0.3]
	[17-18)	1470	57.6 [51.4- 64.6]	-0.3 [-1.1- 0.4]	365	57.7 [52.1-64.2]	-0.3 [-1-0.2]	-0.4 [-1.1-0.2]

Schlüter DK, *et al. Thorax* 2022; 77:136–142. doi: 10.1136/thoraxjnl-2021-216849

Table 8: Height

		US			UK			
birth cohort	age	N	cm	z-score (CDC)	N	cm	z-score (CDC)	z-score (UK WHO)
1985 - 1996	[0-1)	2780	63.0 [59.1- 66.3]	-1.1 [-1.9- -0.2]	6	74.6 [64.4-76]	0.1 [-0.3-0.6]	-0.6 [-1.6-0.2]
	[1-2)	3080	79.0 [75.9- 81.9]	-0.7 [-1.4- 0.0]	21	76.3 [73.6-83.8]	-0.1 [-0.4-0.4]	-0.5 [-1.1-0]
	[2-3)	3452	88.3 [85.8- 91.0]	-0.6 [-1.2- 0.1]	50	88.5 [85.4-92.4]	-0.2 [-0.7-0.3]	-0.6 [-1.2-0]
	[3-4)	3638	95.5 [92.9- 98.3]	-0.6 [-1.3- 0.1]	105	97 [94-99.8]	-0.2 [-0.8-0.3]	-0.7 [-1.3–0.2]
	[4-5)	3741	102.8 [99.7- 105.5]	-0.5 [-1.2- 0.1]	188	102.8 [100-106.2]	-0.4 [-0.8-0.3]	-0.7 [-1.2-0]
	[5-6)	3954	109.0 [105.6- 112.0]	-0.6 [-1.2- 0.1]	311	109.7 [106.6-113.2]	-0.3 [-0.9-0.3]	-0.6 [-1.2-0.1]
	[6-7)	4116	114.8 [111.4- 118.3]	-0.7 [-1.3- 0.0]	431	116 [112.6-119.2]	-0.4 [-1-0.3]	-0.6 [-1.2-0.1]
	[7-8)	4249	120.6 [116.8- 124.3]	-0.8 [-1.4- -0.1]	563	121.7 [118-125.7]	-0.5 [-1.1-0.2]	-0.6 [-1.2-0.1]
	[8-9)	4308	125.9 [122.0- 130.0]	-0.8 [-1.4- -0.1]	694	127 [123-131.2]	-0.6 [-1.2-0.1]	-0.7 [-1.3-0]
	[9-10)	4373	131.0 [127.0- 135.3]	-0.8 [-1.4- -0.1]	766	132.3 [128-136.7]	-0.5 [-1.2-0.2]	-0.6 [-1.2-0.1]
	[10-11)	4420	136.3 [131.9- 140.9]	-0.7 [-1.3- 0.0]	885	137.7 [132.8-142]	-0.4 [-1.2-0.2]	-0.5 [-1.3-0.1]
	[11-12)	4469	141.6 [137.0- 146.7]	-0.7 [-1.4- 0.0]	1002	142.5 [137.8-147.7]	-0.5 [-1.2-0.2]	-0.6 [-1.2-0.2]
	[12-13)	4488	147.5 [142.3- 152.9]	-0.8 [-1.5- -0.1]	1098	148.5 [143.5-153.9]	-0.6 [-1.3-0.1]	-0.5 [-1.2-0.2]
	[13-14)	4522	153.3 [147.9- 159.0]	-0.8 [-1.5- -0.1]	1172	154 [148.2-159.6]	-0.7 [-1.5-0.1]	-0.6 [-1.3-0.2]
	[14-15)	4518	158.3 [152.9- 163.9]	-0.8 [-1.5- 0.0]	1285	158.9 [153.8-164.8]	-0.7 [-1.4-0.1]	-0.6 [-1.4-0.1]
	[15-16)	4526	161.9 [156.3- 168.0]	-0.7 [-1.4- 0.0]	1333	162.3 [156.6-168.5]	-0.6 [-1.3-0.1]	-0.6 [-1.3-0.1]
	[16-17)	4521	164.0 [158.0- 170.5]	-0.6 [-1.3- 0.1]	1624	165 [158.2-171.2]	-0.6 [-1.2-0.1]	-0.7 [-1.3-0]
	[17-18)	4497	165.5 [158.9- 172.3]	-0.6 [-1.2- 0.1]	1625	165.8 [159-172]	-0.6 [-1.2-0.1]	-0.7 [-1.3-0]
1997-2008	[0-1)	3159	63.5 [61.1- 66.0]	-0.9 [-1.7- -0.2]	394	69.4 [61-73]	0 [-0.6-0.7]	-0.8 [-1.8–0.1]
	[1-2)	3570	79.0 [76.5- 81.2]	-0.6 [-1.3- 0.0]	687	78.9 [75.8-83]	-0.1 [-0.8-0.6]	-0.5 [-1.4-0.2]
	[2-3)	3789	88.7 [86.4- 91.3]	-0.4 [-1.1- 0.2]	960	89 [85.7-91.9]	-0.2 [-0.8-0.5]	-0.6 [-1.3-0.1]
	[3-4)	3954	96.3 [93.7- 99.1]	-0.4 [-1.1- 0.3]	996	96.5 [93.4-100]	-0.2 [-0.8-0.5]	-0.7 [-1.3-0]
	[4-5)	4077	103.2 [100.2- 106.2]	-0.4 [-1.1- 0.3]	1095	103.3 [100.2-106.8]	-0.2 [-0.8-0.5]	-0.4 [-1.1-0.2]
	[5-6)	4159	109.6 [106.4- 112.9]	-0.4 [-1.1- 0.2]	1149	109.9 [106.7-113.5]	-0.2 [-0.9-0.4]	-0.5 [-1.2-0.2]
	[6-7)	4247	115.6 [112.3- 119.2]	-0.5 [-1.2- 0.2]	1198	116.1 [113-120]	-0.3 [-0.9-0.3]	-0.4 [-1.1-0.2]
	[7-8)	4296	121.5 [117.8- 125.4]	-0.6 [-1.2- 0.1]	1174	122.1 [118.3-126]	-0.4 [-1-0.3]	-0.4 [-1.1-0.2]
	[8-9)	4311	127.0 [123.3- 131.2]	-0.6 [-1.2- 0.1]	1225	127.9 [124-132]	-0.4 [-1-0.3]	-0.4 [-1.1-0.2]
	[9-10)	4319	132.4 [128.3- 136.8]	-0.5 [-1.2- 0.2]	1148	133.2 [129-137.3]	-0.3 [-1-0.3]	-0.4 [-1.1-0.3]
	[10-11)	4053	137.7 [133.3- 142.4]	-0.5 [-1.1- 0.2]	1086	138.6 [134-142.9]	-0.3 [-1-0.4]	-0.4 [-1.1-0.3]
	[11-12)	3732	143.2 [138.4- 148.5]	-0.5 [-1.1- 0.3]	1026	143.8 [139.1-148.7]	-0.3 [-1-0.3]	-0.3 [-1-0.3]
	[12-13)	3383	149.2 [144.0- 154.8]	-0.6 [-1.3- 0.2]	923	149.6 [144.4-155.2]	-0.4 [-1.2-0.3]	-0.3 [-1.1-0.4]
	[13-14)	3046	154.7 [149.1- 160.3]	-0.6 [-1.4- 0.2]	795	155.1 [149.2-160.7]	-0.5 [-1.3-0.2]	-0.3 [-1.1-0.3]
	[14-15)	2687	158.9 [153.6- 164.9]	-0.6 [-1.4- 0.1]	700	160.1 [154.1-166]	-0.5 [-1.3-0.2]	-0.5 [-1.2-0.3]
	[15-16)	2320	162.1 [156.6- 168.4]	-0.6 [-1.3- 0.1]	586	163.1 [157.4-169.4]	-0.5 [-1.2-0.2]	-0.5 [-1.3-0.2]
	[16-17)	1906	164.2 [158.0- 170.6]	-0.6 [-1.3- 0.1]	498	166.1 [159.2-172]	-0.5 [-1.2-0.3]	-0.6 [-1.3-0.2]
	[17-18)	1470	165.2 [158.6- 171.7]	-0.6 [-1.2- 0.1]	365	166.5 [160.1-172.8]	-0.5 [-1.3-0.2]	-0.7 [-1.4-0.1]

Schlüter DK, *et al. Thorax* 2022; 77:136–142. doi: 10.1136/thoraxjnl-2021-216849

Table 9: BMI

		US			UK			
birth cohort	age	N	kg/cm^2	z-score (CDC)	N	kg/cm^2	z-score (CDC)	z-score (UK WHO)
1985 - 1996	[0-1)	2780	15.1 [13.9- 16.3]	N|A	6	16 [15.9-16.9]	NA [NA-NA]	-0.6 [-1.1-0.4]
	[1-2)	3080	16.5 [15.6- 17.5]	N|A	21	16.9 [15.5-18.3]	NA [NA-NA]	0.6 [-0.3-1.4]
	[2-3)	3452	16.2 [15.4- 17.1]	-0.01 [-0.7- 0.6]	50	16 [14.9-17]	-0.1 [-1.1-0.7]	0.3 [-0.5-1.1]
	[3-4)	3638	15.8 [15.1- 16.7]	0.1 [-0.5- 0.7]	105	16.1 [15.1-16.9]	0.3 [-0.4-0.9]	0.5 [-0.2-1.1]
	[4-5)	3741	15.6 [14.8- 16.4]	0.1 [-0.5- 0.7]	188	15.7 [14.8-16.6]	0.3 [-0.6-0.9]	0.1 [-0.7-0.7]
	[5-6)	3954	15.4 [14.7- 16.2]	0.1 [-0.5- 0.7]	311	15.6 [14.8-16.7]	0.3 [-0.4-0.9]	0.1 [-0.5-0.8]
	[6-7)	4116	15.4 [14.6- 16.3]	0.001 [-0.6- 0.5]	431	15.5 [14.7-16.4]	0.1 [-0.5-0.7]	0 [-0.6-0.6]
	[7-8)	4249	15.5 [14.7- 16.4]	-0.1 [-0.6- 0.4]	563	15.7 [14.9-16.9]	0.1 [-0.4-0.7]	0 [-0.5-0.7]
	[8-9)	4308	15.7 [14.9- 16.7]	-0.2 [-0.7- 0.4]	694	15.9 [15-17.1]	0 [-0.6-0.6]	-0.1 [-0.6-0.6]
	[9-10)	4373	16.0 [15.1- 17.1]	-0.2 [-0.8- 0.3]	766	16.3 [15.3-17.5]	-0.1 [-0.7-0.5]	-0.1 [-0.7-0.6]
	[10-11)	4420	16.4 [15.4- 17.7]	-0.3 [-0.8- 0.3]	885	16.6 [15.6-18.1]	-0.1 [-0.7-0.4]	-0.1 [-0.7-0.5]
	[11-12)	4469	16.8 [15.8- 18.2]	-0.4 [-0.9- 0.2]	1002	17.1 [15.9-18.7]	-0.2 [-0.8-0.4]	-0.2 [-0.8-0.5]
	[12-13)	4488	17.3 [16.2- 18.9]	-0.4 [-1.0- 0.2]	1098	17.8 [16.3-19.3]	-0.2 [-0.9-0.4]	-0.1 [-0.9-0.5]
	[13-14)	4522	18.1 [16.7- 19.7]	-0.3 [-1.0- 0.3]	1172	18.3 [16.8-19.9]	-0.2 [-0.9-0.3]	-0.2 [-0.9-0.5]
	[14-15)	4518	18.9 [17.4- 20.5]	-0.3 [-0.9- 0.3]	1285	18.9 [17.5-20.6]	-0.2 [-0.9-0.3]	-0.2 [-0.8-0.5]
	[15-16)	4526	19.6 [18.1- 21.2]	-0.2 [-0.9- 0.3]	1333	19.6 [18.1-21.3]	-0.2 [-0.8-0.4]	-0.2 [-0.8-0.5]
	[16-17)	4521	20.1 [18.6- 21.7]	-0.3 [-0.9- 0.3]	1624	20.1 [18.6-21.8]	-0.2 [-0.9-0.3]	-0.1 [-0.8-0.5]
	[17-18)	4497	20.4 [18.9- 22.1]	-0.3 [-1.0- 0.2]	1625	20.2 [18.6-21.9]	-0.4 [-1.1-0.2]	-0.3 [-1-0.4]
1997-2008	[0-1)	3159	15.7 [14.8- 16.7]	N|A	394	16.8 [15.2-18]	NA [NA-NA]	0 [-1-0.9]
	[1-2)	3570	16.7 [15.9- 17.6]	N|A	687	17.1 [16.1-18.1]	NA [NA-NA]	0.7 [0.1-1.4]
	[2-3)	3789	16.3 [15.5- 17.1]	0.1 [-0.6- 0.7]	960	16.6 [15.7-17.5]	0.2 [-0.4-0.9]	0.7 [0-1.3]
	[3-4)	3954	15.9 [15.2- 16.7]	0.2 [-0.4- 0.8]	996	16.2 [15.4-17]	0.4 [-0.3-1]	0.6 [0-1.2]
	[4-5)	4077	15.7 [14.9- 16.4]	0.2 [-0.4- 0.7]	1095	15.9 [15.1-16.8]	0.4 [-0.3-1]	0.2 [-0.4-0.8]
	[5-6)	4159	15.5 [14.9- 16.3]	0.2 [-0.4- 0.7]	1149	15.6 [14.9-16.4]	0.2 [-0.3-0.8]	0.1 [-0.5-0.6]
	[6-7)	4247	15.6 [14.9- 16.5]	0.1 [-0.4- 0.6]	1198	15.6 [14.8-16.6]	0.2 [-0.4-0.7]	0.1 [-0.5-0.6]
	[7-8)	4296	15.8 [15.0- 16.8]	0.1 [-0.4- 0.6]	1174	15.8 [14.9-16.8]	0.1 [-0.5-0.7]	0 [-0.6-0.6]
	[8-9)	4311	16.1 [15.3- 17.2]	0.05 [-0.5- 0.6]	1225	16 [15.1-17.3]	0 [-0.5-0.6]	0 [-0.6-0.7]
	[9-10)	4319	16.5 [15.6- 17.8]	-0.004 [-0.5- 0.5]	1148	16.4 [15.4-17.7]	0 [-0.6-0.6]	0 [-0.6-0.7]
	[10-11)	4053	16.9 [15.9- 18.3]	-0.1 [-0.6- 0.5]	1086	16.8 [15.7-18.2]	-0.1 [-0.6-0.5]	0 [-0.7-0.6]
	[11-12)	3732	17.4 [16.3- 18.8]	-0.1 [-0.6- 0.4]	1026	17.3 [16.2-18.9]	-0.1 [-0.7-0.5]	0 [-0.7-0.6]
	[12-13)	3383	18.0 [16.7- 19.5]	-0.1 [-0.7- 0.4]	923	17.8 [16.6-19.4]	-0.1 [-0.7-0.5]	-0.1 [-0.7-0.6]
	[13-14)	3046	18.7 [17.4- 20.4]	-0.1 [-0.7- 0.5]	795	18.5 [17.1-20.2]	-0.1 [-0.7-0.5]	-0.1 [-0.7-0.6]
	[14-15)	2687	19.4 [18.0- 21.2]	-0.04 [-0.6- 0.5]	700	19.2 [17.7-20.8]	-0.1 [-0.8-0.4]	0 [-0.8-0.6]
	[15-16)	2320	20.1 [18.6- 21.9]	-0.02 [-0.6- 0.5]	586	19.8 [18.3-21.5]	-0.1 [-0.8-0.5]	0 [-0.7-0.6]
	[16-17)	1906	20.7 [19.0- 22.5]	-0.04 [-0.7- 0.5]	498	20.5 [18.9-22.3]	-0.1 [-0.7-0.5]	0 [-0.7-0.7]
	[17-18)	1470	21.0 [19.3- 22.9]	-0.1 [-0.8- 0.5]	365	21 [19.3-22.6]	-0.1 [-0.8-0.4]	0 [-0.7-0.6]

Schlüter DK, *et al. Thorax* 2022; 77:136–142. doi: 10.1136/thoraxjnl-2021-216849

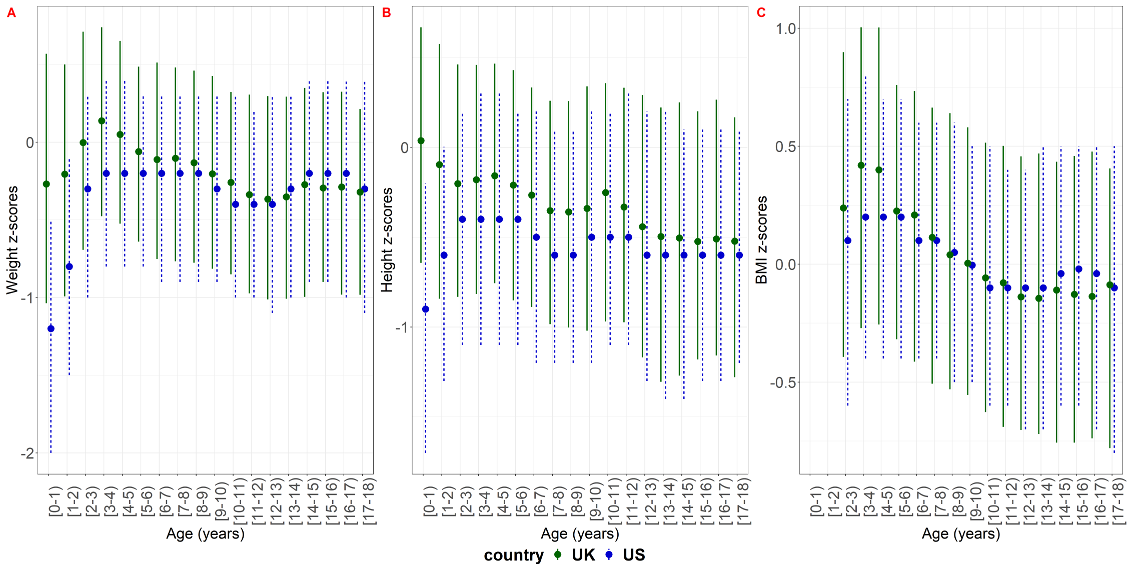

Figure 13: Cross-sectional summary measures of weight, height and BMI z-scores of the population born after 1997 based on the CDC reference population in the US and the UK. Panels A-C show the median, 25th and 75th percentiles The dots show the median and the lines the interquartile range. Plots for the whoel population are given in the main article.

Schlüter DK, *et al. Thorax* 2022; 77:136–142. doi: 10.1136/thoraxjnl-2021-216849

10. Treatments over period from first registry recording to <18 years

In tables 10-15 we present the proportion of individuals with at least one record of receiving the treatments before age 6, before age 12 and before age 18; as well as the median, 25th and 75th percentile, minimum and maximum of the age at first recorded treatment. This may include records collected prior to 2003 but observations remained censored at transplant. We stratified these outcomes by year of birth (before and after 1997) as post 1997 data will be more complete with regards to treatments and may give a better representation of healthcare practices.

Note: For treatements and infections we calculate the proportion of individuals who have a recording for treatment or infection out of those individuals in the cohort with at least one recording in the registry during that age range.

For the 1985-1996 cohort we observed 325 under the age of 6 and 1150 under the age of 12 out of 1601 overall in the UK.
For the 1997-2008 cohort we observed 1256 under the age of 6 and 1423 under the age of 12 out of 1454 overall in the UK.

In the US we observed 4264 individuals under the age of 6 and 4730 under the age of 12 out of 4889 overall for the 1985-1996 cohort.
For the 1997-2008 cohort we observed 4266 individuals before the age of 6 and 4533 under the age of 12 out of 4572 overall.

Table 10: DNase

	US proportion treated			US age at first recorded treatment			UK proportion treated			UK age at first recorded treatment		
cohort	< age 6	< age 12	< age 18	median[IQR]	min	max	< age 6	< age 12	< age 18	median[IQR]	min	max
1985-1996	0.21	0.72	0.91	8.7 [6.4, 11.5]	0.02	17.98	0.05	0.30	0.65	13.17 [11-15.33]	3.58	17.92
1997-2008	0.73	0.93	0.96	3.9 [1.6, 6.4]	0	16.67	0.20	0.72	0.86	8.58 [6.33-11]	0.25	17.92

Table 11: Hypertonic Saline

	US proportion treated			US age at first recorded treatment			UK proportion treated			UK age at first recorded treatment		
cohort	< age 6	< age 12	< age 18	median[IQR]	min	max	< age 6	< age 12	< age 18	median[IQR]	min	max
1985-1996	0	0.07	0.43	14.8 [12.9, 16.5]	9.06	17.99	0.00	0.01	0.14	16 [14.9-16.92]	11.08	17.92
1997-2008	0.24	0.68	0.76	7.8 [5.7, 10.0]	0	17.74	0.04	0.33	0.50	10.83 [8.08-13]	2.00	17.92

Table 12: Tobramycin

	US proportion treated			US age at first recorded treatment			UK proportion treated			UK age at first recorded treatment		
cohort	< age 6	< age 12	< age 18	median[IQR]	min	max	< age 6	< age 12	< age 18	median[IQR]	min	max
1985-1996	0.18	0.65	0.89	9.7 [6.9, 12.5]	0.12	17.98	0.02	0.08	0.26	14.83 [12.17-16.5]	4.08	17.92
1997-2008	0.62	0.77	0.8	3.5 [1.5, 6.4]	0	17.39	0.06	0.22	0.33	10.17 [7.21-13]	0.83	17.92

Table 13: Colistin/ Promixin/ Colistimethate

	US proportion treated			US age at first recorded treatment			UK proportion treated			UK age at first recorded treatment		
cohort	< age 6	< age 12	< age 18	median[IQR]	min	max	< age 6	< age 12	< age 18	median[IQR]	min	max
1985-1996	0	0.01	0.12	15.1 [13.3, 16.6]	9.11	17.99	0.45	0.72	0.87	10.83 [8.17-13.67]	1.17	17.92
1997-2008	0.02	0.07	0.09	9.1 [6.7, 12.2]	0.22	16.64	0.51	0.77	0.83	4.67 [2.75-8.17]	0.00	17.92

Schlüter DK, *et al. Thorax* 2022; 77:136–142. doi: 10.1136/thoraxjnl-2021-216849

Table 14: Aztreonam

	US proportion treated			US age at first recorded treatment			UK proportion treated			UK age at first recorded treatment		
cohort	< age 6	< age 12	< age 18	median[IQR]	min	max	< age 6	< age 12	< age 18	median[IQR]	min	max
1985-1996	0	0	0.12	16.4 [15.4, 17.3]	9.81	17.99	0	0	0.00	17.08 [16.79-17.88]	15.92	17.92
1997-2008	0.02	0.14	0.24	11.4 [9.0, 13.3]	1.88	17.95	0	0	0.02	14.58 [13.02-16.27]	10.17	17.92

Table 15: Colistin, Tobramycin or Aztreonam

	US proportion treated			UK proportion treated		
cohort	< age 6	< age 12	< age 18	< age 6	< age 12	< age 18
1985-1996	0.18	0.65	0.89	0.46	0.73	0.88
1997-2008	0.62	0.77	0.8	0.51	0.78	0.84

Schlüter DK, *et al. Thorax* 2022; 77:136–142. doi: 10.1136/thoraxjnl-2021-216849

11. Infections over period from first registry recording to <18 years

In tables 16 and 17 we present the proportion of individuals with at least one record of having an infection before age 6, before age 12 and before age 18; as well as the median, 25th and 75th percentile, minimum and maximum of the age at first recorded infection. This may include records collected prior to 2003 but observations remained censored at transplant. We stratified these outcomes by year of birth (before and after 1997) as post 1997 data will be more complete with regards to treatments and infections and may give a better representation of healthcare practices.
We calculate the proportion of individuals who have a recording for an infection out of those individuals in the cohort with at least one recording in the registry during that age range.

In the UK any infections since the last annual review visit are recorded in annual review. Therefore the number of children with information on microbiology is the same as the the total number observed at any particular age (see section on treatments).
In the US, microbiology was not necessarily assessed at each encounter, therefore the number of people with an encounter visit at which treaments are recorded, may differ from the number of people with an encounter visit at which also mircrobiology samples are taken. For the 1985-1996 cohort we observed 4229 under the age of 6, 4742 under the age of 12 and 4887 under the age 18 had at least mircobiology sample taken.
For the 1997-2008 cohort we observed 4275 under the age of 6, 4533 under the age of 12 and 4572 under the age 18 had at least mircobiology sample taken.

Table 16: Pseudomonas

	US proportion with recorded infection			US age at first recorded infection			UK proportion with recorded infection			UK age at first recorded infection		
cohort	< age 6	< age 12	< age 18	median[IQR]	min	max	< age 6	< age 12	< age 18	median[IQR]	min	max
1985-1996	0.58	0.81	0.93	5.4 [2.3, 9.5]	0	17.98	0.4	0.67	0.89	11.42 [8.83-14.08]	1.08	17.92
1997-2008	0.71	0.84	0.86	2.6 [1.1, 5.7]	0	17.73	0.6	0.82	0.89	4.92 [2.5-8.83]	0.00	17.58

Table 17: Methicillin sensitive (MSSA) and methicillin resistant (MRSA) Staphylococcus aureus

		US proportion with recorded infection			US age at first recorded infection			UK proportion with recorded infection			UK age at first recorded infection		
	cohort	< age 6	< age 12	< age 18	median[IQR]	min	max	< age 6	< age 12	< age 18	median[IQR]	min	max
MSSA	1985-1996	0.68	0.91	0.96	4.5 [1.9, 8.0]	0	17.97	0.28	0.57	0.74	11.33 [8.67-14.33]	1.00	17.92
	1997-2008	0.87	0.95	0.96	2.1 [0.6, 4.4]	0	16.93	0.43	0.70	0.78	6.08 [3.42-9.17]	0.00	17.92
MRSA	1985-1996	0.03	0.19	0.43	12.8 [9.8, 15.5]	0.11	17.99	0.00	0.04	0.07	13 [9.96-15.88]	6.58	17.92
	1997-2008	0.27	0.5	0.55	6.5 [3.7, 9.2]	0	17.57	0.03	0.07	0.10	8.54 [6-12.31]	0.50	17.92

Schlüter DK, *et al. Thorax* 2022; 77:136–142. doi: 10.1136/thoraxjnl-2021-216849

12. Results from robustness test

To assess the generalisability of our results to more recent the current paediatric CF populations, we repeated our analysis using re-fitted the best fitting models applied to the recent cohort born after 1997.
We found that in both countries lung function decline was less steep in the population born after 1997 compared to the whole study population. Based on the model that included a linear function of age, the US mean lung function decline in the post-1997 cohort was -1.18 percentage points per year (95% CI: -1.25 to -1.1), in the UK it was -1.34 percentage points per year (95% CI: -1.5 to -1.19). Children in the UK population were estimated to lose an additional 0.17 percentage points of FEV1 per year (95% CI -0.004 to 0.34) compared to US children (Figure 14). The estimated gap in absolute level of lung function was comparable to the estimates derived from the whole population. Based on the model that included a linear function of age and at reference covariate values, the gap was estimated to be 4.52 (95% CI 2.34 to 6.69), 5.52 (95% CI 3.06 to 7.97) and 6.35 (95% CI 3.39 to 9.3) percentage points of FEV1 at ages 6, 12 and 17, respectively. The estimates based on the model that included age using a spline with 5 knots were 4.72 (95% CI 2.35 to 7.09), 5.21 (95% CI 3.04 to 7.39) and 4.33 (1.73 to 6.94) percentage points FEV1.

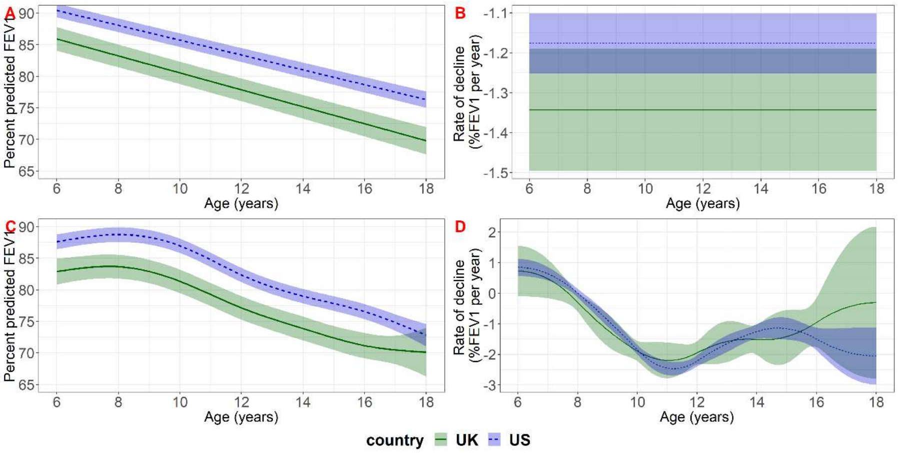

Figure 14: Estimated population-level mean lung function (panels A and C) and lung function decline (panels B and D) in the UK and US study populations born after 1997 for reference covariate values (female, born in 1997, diagnosed at birth). The top row (panels A and B) shows results based on the model hat included a linear function of age with random intercept and slope and exponential correlation function; the bottom row (panels C and D) shows results based on the model that included age using a spline with 5 knots with random intercept and exponential correlation function.

Schlüter DK, *et al. Thorax* 2022; 77:136–142. doi: 10.1136/thoraxjnl-2021-216849

13. Comparison of annual(UK) and average(US) lung function in 2014 with best measurement

Previously it has been reported that there is a significant difference between annual and best %FEV1 measurement in the UK. Data on best %FEV1 have only been collected in the UK CF Registry since 2012 and converage was initially poor. The best coverage for the period under study was in 2014. To assess whether the difference between annual and best measure could explain our findings, we used the data collected in 2014 and estimated 1) the mean of the average %FEV1 across all encounters per person in the US 2) the mean of the best %FEV1 measures in the US 3) the mean of the annual review %FEV1 in the UK and 4) the mean of the best %FEV1 in the UK. We present the results stratified by age. In the UK, best FEV1 is recorded in the Registry at annual review and is therefore the best measure since the last annual review. It may therefore be a measurement from another year and/or when the individual was younger. In the US, we extracted the best FEV1 measurement for each individual in 2014 and calculated the average of all encounters at which the individual was the same age that they were when the best FEV1 was measured. The table below shows that the difference between annual and best in the UK is closely mirrored by the difference between average and best in the US.

Table 18: Mean of the 1) average %FEV1 across all encounters per person in the US 2) best %FEV1 per person in the US 3) annual review %FEV1 per person in the UK and 4) best %FEV1 per person in the UK in 2014 stratified by age at the end of the year (US) and at annual review (UK).

	US			UK			
age	N	mean best %FEV1 (sd)	mean average %FEV1 (sd)	N	% with best %FEV1 measure	mean best %FEV1 (sd)	mean annual %FEV1 (sd)
6	410	100.1 (16.6)	94.8 (16.6)	114	91	94.07 (14.68)	89.52 (17.07)
7	367	103.9 (16.9)	96.9 (17.2)	124	84	92.17 (13.95)	89.25 (16.01)
8	345	101.7 (16.6)	94.9 (17.2)	111	77	92.12 (13.94)	87.64 (16.59)
9	365	100.9 (15.7)	94.9 (16.4)	127	80	91.74 (14.24)	88.57 (15.44)
10	352	97.4 (15.4)	91.6 (16.3)	122	84	89.05 (13.92)	84.59 (16.33)
11	354	95.3 (17.2)	89.2 (17.9)	120	82	85.59 (15.28)	83.03 (17.19)
12	420	92.4 (18.1)	86.4 (19.1)	103	77	82.2 (16.29)	81.67 (16.62)
13	379	92.1 (19.1)	86.0 (19.5)	124	82	80.96 (17.1)	77.34 (18.46)
14	385	88.3 (18.1)	82.2 (19.0)	110	72	81.49 (17.62)	77.59 (18.49)
15	394	88.1 (20.0)	81.6 (20.2)	135	79	81.66 (20.28)	74.7 (22.43)
16	380	87.4 (22.1)	80.9 (22.9)	106	82	79.61 (17.15)	74.89 (18.44)
17	505	84.0 (21.0)	78.2 (21.0)	151	66	77.42 (19.51)	71.46 (21.1)

Schlüter DK, *et al. Thorax* 2022; 77:136–142. doi: 10.1136/thoraxjnl-2021-216849
